# PARPi Combining Nanoparticle LIN28B siRNA for the Management of Malignant Ascites

**DOI:** 10.1002/advs.202510547

**Published:** 2026-01-22

**Authors:** Yan Fang, Qian Shen, Yao Lin, Jing Zhu, Xiaolan Zhu, Rui Huang, Yijia Wu, Feiyang Shen, Qian Li, Guopei Zheng, Zhe Zhang, Qian Chu, Junhao Hu, Jianfeng Shen

**Affiliations:** ^1^ Department of Ophthalmology, Ninth People's Hospital Shanghai Jiao Tong University School of Medicine Shanghai China; ^2^ Shanghai Key Laboratory of Orbital Diseases and Ocular Oncology Shanghai China; ^3^ Institute of Translational Medicine National Facility for Translational Medicine Shanghai Jiao Tong University Shanghai China; ^4^ Department of Oncology, Tongji Hospital Tongji Medical College Huazhong University of Science and Technology Wuhan China; ^5^ Department of Gastrointestinal Surgery Union Hospital Tongji Medical College Huazhong University of Science and Technology Wuhan China; ^6^ Interdisciplinary Research Center on Biology and Chemistry Shanghai Institute of Organic Chemistry Chinese Academy of Sciences University of Chinese Academy of Sciences Beijing China

**Keywords:** malignant ascites, nanoparticle LIN28B siRNA, neutrophil, PARP inhibitor, vascular permeability

## Abstract

Malignant serous effusion (MSE), including malignant pleural effusion (MPE) and malignant ascites (MA), is a common and severe complication in advanced malignancies, associated with poor prognosis and high recurrence rates. Currently, no standardized treatments are available for MSE management, posing significant clinical challenges. Here, we identify elevated LIN28B expression and dysregulation of DNA repair pathways as two major features associated with MSE from patient and preclinical samples. We develop a targeted siRNA nanoparticle delivery system (siLin28B/DSSP@lip‐PEG‐FA) in combination with the PARP inhibitor BMN673, providing a synergistic therapeutic strategy against MSE. This combination significantly alleviated MA accumulation and prolonged survival in a preclinical ovarian cancer (OC) model without causing systemic cytotoxicity. Mechanistically, single‐cell RNA sequencing (scRNA‐seq) revealed that this combination therapy markedly remodeled the immune microenvironment by decreasing M2 macrophages and neutrophil populations with altered subtypes. Notably, Arg1‐positive neutrophils, producing pro‐inflammatory cytokines to increase vascular permeability, were diminished after the combination treatment. Furthermore, in vitro and in vivo experiments demonstrated that suppression of PARP and LIN28B inhibited vascular leakage and reinforced tight junction integrity. Collectively, our findings highlight dual targeting of PARP and LIN28B as a promising MA management approach in patients with advanced cancers, with the potential to improve patient quality of life.

## Graphical abstract

This study demonstrates that co‐inhibition of LIN28B and PARP using siLin28b/DSSP@lip‐PEG‐FA nanoparticles in combination with the PARP inhibitor BMN673 effectively suppresses the accumulation of malignant ascites associated with advanced cancers. This therapeutic strategy improves vascular integrity, reduces infiltration of immunosuppressive macrophages, and attenuates the secretion of pro‐tumorigenic cytokines.

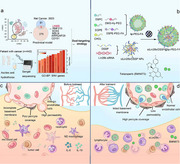

**Abbreviations**
ALT: aspartate alanine aminotransferase
AST: aspartate aminotransferase
BUN: blood urea nitrogen
CEACAM: Carcinoembryonic Antigen‐Related Cell Adhesion Molecule
CLSM: confocal laser scanning microscopy
CNVs: copy number variations
CopyKAT: Copy number Karyotyping of Aneuploid Tumors
CREA: creatinine
DEGs: differentially expressed genes
GO: Gene Ontology
GO‐BP: Gene Ontology Biological Process
GSEA: gene set enrichment analysis
GSH: glutathione
H_2_O_2_
: hydrogen peroxide
IR: infrared
MA: malignant ascites
MPE: malignant pleural effusion
MSE: Malignant serous effusion
NGS: Next‐generation sequencing
OC: ovarian cancer
PBS: phosphate‐buffered saline
scRNA‐seq: single‐cell RNA sequencing
SNVs: single nucleotide variations
TBIL: total bilirubin
TCBA: thiocystamine bisacrylamide
UA: uric acid
VEGF: vascular endothelial growth factor

## 1 Introduction

Malignant serous effusion (MSE) is characterized by the abnormal accumulation of fluid within the pleural, peritoneal, or pericardial cavities, and commonly arises from invasion of primary tumors into body cavities and serous membranes, leading to local metastasis and systemic dissemination [1, 2]. Among MSE subtypes, malignant pleural effusion (MPE) is most frequently associated with lung cancer (37.5%), breast cancer (16.8%), and malignant lymphoma (11.5%), whereas malignant ascites (MA) is predominantly observed in ovarian cancer (OC) (37%), hepatobiliary and pancreatic tumors (21%), and gastric cancer (18%) [3, 4]. MA, arising from malignant peritoneal metastases, indicates an advanced stage of disease and is often life‐threatening, with a poor prognosis and high recurrence rates [5, 6]. Management of MA poses significant clinical challenges, particularly for patients who are not candidates for surgical intervention [7, 8, 9]. These unmet clinical needs underscore the urgency of developing effective therapeutic strategies to improve patient quality of life.

Current standard therapies for MA include paracentesis, diuretics, peritoneovenous shunting, permanent drainage catheters, and intracavitary chemotherapies [10]. However, few of these treatments provide durable efficacy with acceptable safety profiles [11]. For instance, serial paracenteses can temporarily drain intraperitoneal fluid, but symptoms typically recur within weeks, with an increased risk of infection [12]. Diuretic therapy remains controversial, as many physicians consider it ineffective [13, 14]. Peritoneovenous shunting requires perioperative hospitalization and carries risks such as shunt occlusion, infection, bleeding, and even death [14]. Intraperitoneal chemotherapies, although commonly used, lack specificity for cancer cells, resulting in low response rates and high incidences of side effects [10]. Hyperthermic intraperitoneal chemotherapies, though widely adopted, are palliative rather than curative [15, 16]. To address the urgent need for novel therapeutic strategies, it is critical to elucidate the molecular features and mechanisms underlying MSE.

Recent studies have revealed distinct molecular and functional changes in MA, including alterations in immune cell composition and metabolic profiles in advanced malignancies such as gastric and ovarian cancers [17, 18]. Snijder et al. comprehensively characterized the genomic and transcriptomic features of 150 MSE samples (mostly MPE and MA), identifying gene expression patterns associated with drug sensitivity and potential therapeutic targets [19]. Despite these advances, larger datasets are needed to establish links between the molecular characteristics of MA cells, particularly cancer cells, and effective therapeutic strategies. In this study, we analyzed a cohort of 442 MSE samples to identify genomic features and integrated these data with gene expression patterns from preclinical and clinical samples. Our results indicate that dual inhibition of PARP and LIN28B represents a promising strategy for MA management, with potential for further clinical development.

## 2 Results

### 2.1 Aberrant LIN28B Expression and DNA Repair Pathways are Associated with MSE

First, we used a syngeneic OC mouse model to study MSE. Forty‐five days after intraperitoneal injection of murine ovarian cancer ID8 cells (1×10^7^), we collected peritoneal, mesenteric, and relevant tissues from both tumor‐bearing and healthy C57BL/6 mice (Figure S1a). Notably, tumor‐bearing mice developed substantial volumes of malignant ascitic fluid, accompanied by compromised mesenteric vasculature, as evidenced by Evans blue perfusion assays (Figure S1b). Immunohistochemical analysis further revealed pronounced vascular leakage in both the peritoneum and mesentery of tumor‐bearing mice, as indicated by the extravasation of Ter119^+^ erythroid cells from CD31^+^ vascular networks (Figure S1c), recapitulating key clinical phenotypes of MSE [20].

**FIGURE 1:**
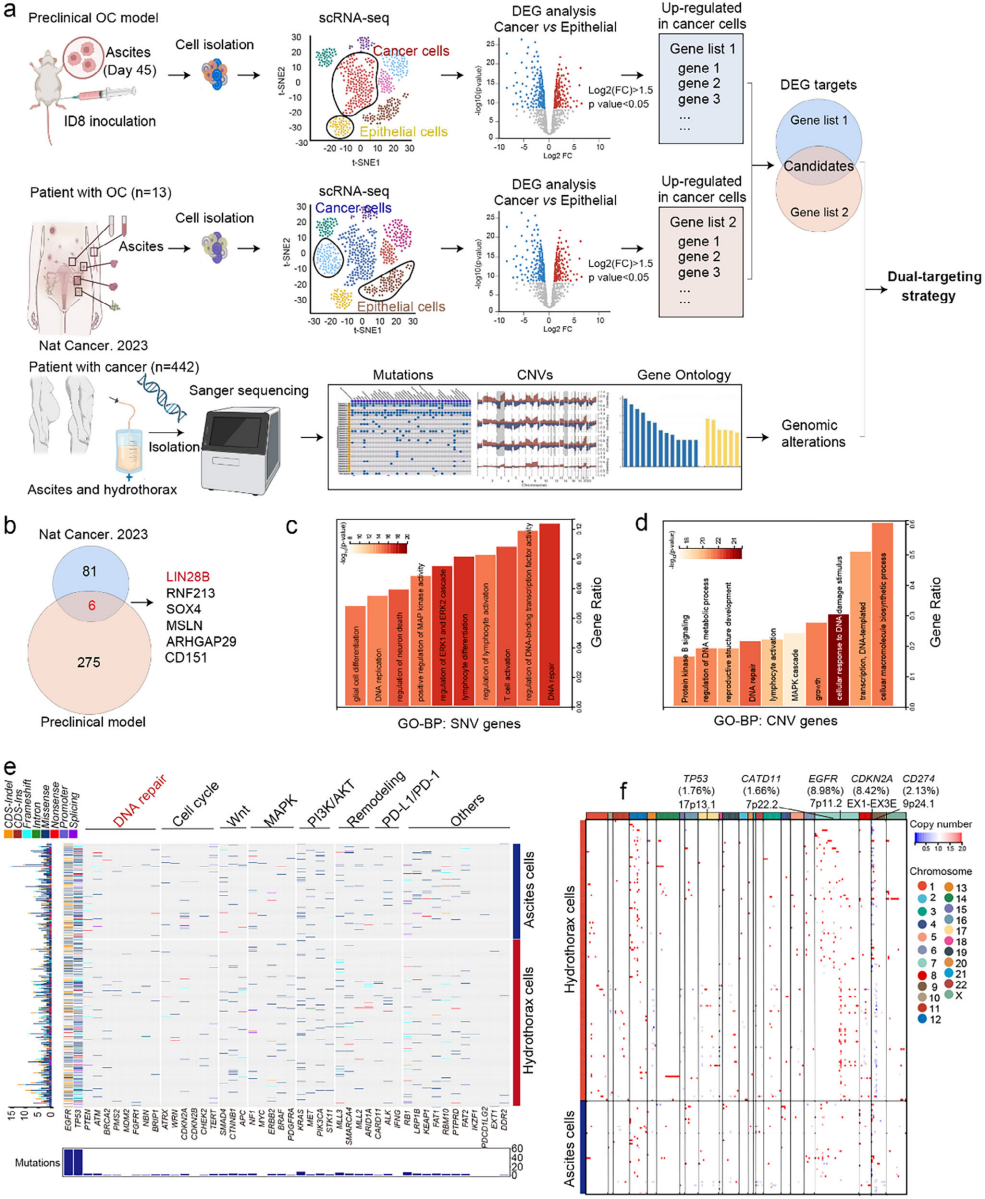
Aberrant LIN28B expression and DNA repair pathway are associate with MSE. (a) Integrated analysis combining scRNA‐seq data from ascites in a constructed preclinical OC model; scRNA‐seq data from MA in OC patients (*n* = 13), and gene mutation profiles from MSE in cancer patients (*n* = 442) to explore novel dual‐targeting therapeutic strategies (created at BioRender.com). (b) Venn diagram analysis of DEG targets between the preclinical OC model and OC patients (Nat Cancer. 2023). GO‐BP enrichment analysis for (c) SNV gene mutations and (d) CNV gene mutations in MSE from cancer patients. (e) Waterfall plot for top 45 SNV genes and relative pathways. Genotype Quality (GQ): Minimum threshold of 20 (99% confidence in the genotype call). Read Depth (DP): Site‐specific depth filters (DP ≥ 10 for high‐confidence calls, adjusted for cohort mean depth). lternate Allele Support: Minimum alternative allele reads (≥ 3) and fraction (AF ≥ 0.25 for heterozygous germline calls). opulation Frequency: Filtering against population databases (gnomAD allele frequency < 0.01 for rare variants, < 0.001 for ultra‐rare). (f) Heatmap of CNV genes in MSE from cancer patients (*n* = 442). Quality Metrics: Segment‐wise log2 ratio standard deviation or confidence score (e.g., CNVkit quality score > 0.5). Size Threshold: Typically, > 1 kb for targeted sequencing and > 10–50 kb for whole‐genome sequencing to exclude small, noisy calls. Copy State Threshold: Log2 ratio thresholds define copy states (e.g., deletion: log2 ratio < −0.4, duplication: log2 ratio > 0.2).

We next investigated the transcriptomic features of MSE cells (Figure 1a). Single‐cell RNA sequencing (scRNA‐seq) was performed on MA cells from ID8 tumor‐bearing mice. In parallel, publicly available scRNA‐seq datasets of MA from ovarian cancer patients were analyzed [10]. To identify actionable targets, we compared differentially expressed genes (DEGs) between cancer cells and epithelial cells in both datasets (Figure 1a). Cross‐comparison of cancer cells DEGs from the preclinical model (275 genes) and OC patient samples (81 genes) identified six shared genes: *LIN28B*, *RNF213*, *SOX4*, *MSLN*, *ARHGAP29*, and *CD151* (Figure 1b). Among these, *LIN28B* was consistently and markedly upregulated in cancer cells relative to non‐malignant populations in both datasets (Figure S1d,e). We further evaluated the enrichment ratios of all six candidates at the single‐cell level, and as shown in Figure S1f, *LIN28B* exhibited the highest specificity. Moreover, analysis of The Cancer Genome Atlas dataset demonstrated that *LIN28B*, unlike the other five genes, displayed a clear tumor‐specific expression pattern, reinforcing its prioritization as a key candidate in this study.

Additionally, we collected 442 MSE samples, including MPE (hydrothorax) and MA (ascites), from patients with multiple cancer types (Table S1). Next‐generation sequencing (NGS) was performed to analyze single nucleotide variations (SNVs), copy number variations (CNVs), and Gene Ontology (GO) terms in these samples (Figure 1a). GO enrichment analysis revealed significant associations of SNV‐ and CNV‐related genes with DNA repair pathways (Figure 1c–e). Among CNVs, the most frequently altered genes were *EGFR* (8.98%), *CDKN2A* (8.42%), *CD274* (2.13%), *CARD11* (1.66%), and *TP53* (1.76%). Notably, CNV deletions were primarily observed in *CDKN2A* and *CD274*, whereas amplifications were enriched in *EGFR* and *TP53* (Figure 1f). Consistent with these results, several DNA repair‐related pathways, such as PI3K‐Akt, MAPK, EGFR tyrosine kinase inhibitor resistance, and p53 signaling, were significantly enriched among genes harboring either SNVs or CNVs (Figure S1g). Collectively, these results indicate that elevated *LIN28B* expression and aberrations in DNA repair pathways are key molecular features associated with MSE, highlighting their potential as therapeutic targets.

### 2.2 Synthesis and Characterization of DSSP@lip‐PEG‐FA Targeting LIN28B

#### 2.2.1 Characterization of DSSP

To therapeutically target elevated *LIN28B* in cancer cells, we designed and synthesized a redox‐responsive nanoparticles incorporating siRNA against *LIN28B*. As illustrated in Figure S2a, the redox‐sensitive polyamine (DSSP) was synthesized via a two‐step reaction. The chemical structure of DSSP was confirmed by infrared (IR) spectroscopy, proton nuclear magnetic resonance (^1^H NMR), and carbon nuclear magnetic resonance (^13^C NMR) (Figure S2b–e).

Analysis of the ^1^H‐NMR spectrum revealed coupled methylene peaks at δ 2.718 (d, J = 6.0 Hz, 2H) and δ 3.536 (d, J = 6.0 Hz, 2H), indicating the presence of the structure ‐S‐CH_2_‐CH_2_‐NH‐ moiety. Additionally, the coupled unsaturated proton signals observed at δ 6.273 (d, J = 16.8 Hz, 1H), δ 6.177 (dd, J = 16.8 Hz and 10.2 Hz, 1H), and δ 5.640 (d, J = 10.2 Hz, 1H) correspond to the vinyl group (–CH = CH_2_). The ^13^C‐NMR spectrum further confirmed the presence of two unsaturated carbons (δ 130.82 and 126.91), two saturated carbons (δ 38.91 and 32.01), and one amide carbon (δ 166.08), collectively validating the structural of thiocystamine bisacrylamide (TCBA) (Figure S2c).

The ‐S‐CH_2_‐CH_2_‐NH‐ signals (δ 3.536 and δ 2.718) closely matched the methylene resonances at δ 3.387 and δ 2.704 observed in Figure S2d. Similarly, the ^13^C chemical shifts at δ 38.91 and δ 32.01 in Figure S2c corresponded well with δ 37.454, δ 31.148 in Figure S2d, confirming the retention of the amide group (δ ∼162–166). The presence of spermine‐derived structural motifs was evidenced by the characteristic methylene resonances at δ 2.619, δ 2.577, δ 2.547, δ 1.595, and δ 1.472 (Figure S2e), which closely matched δ 2.990, δ 2.911, δ 2.849, δ 1.797, and δ 1.639 in Figure S2d. The ^13^C NMR chemical shifts at δ 48.932, δ 46.657, δ 32.026, and δ 26.863 in Figure S2e also aligned with δ 48.412, δ 46.256, δ 27.972, and δ 25.534 in Figure S2d, confirming the presence of the ‐NH‐CH_2_‐CH_2_‐CH_2_‐NH‐CH_2_‐CH_2_‐ fragment derived from spermine in DSSP.

Importantly, the disappearance of vinyl group signals in the final product and the emergence of new methylene peaks, such as the shift from δ 39.146 to δ 45.786 (Figure S2e vs. S2d), indicate a successful addition reaction between TCBA and spermine. Collectively, these data confirm the successful synthesis and structural integrity of DSSP as a backbone for siRNA delivery. Although the proton‐buffering capacity of DSSP was lower than that of PSP‐Control polymer, it remained significantly higher than that of NaCl control (Figure S2f). Cytotoxicity assays further indicated that DSSP exhibited reduced cytotoxicity relative to PSP‐Control, a structurally similar polyamine lacking redox‐sensitive bonds (Figure S2g,h).

#### 2.2.2 Characterization of siRNA/DSSP@lip‐PEG‐FA

To determine the optimal formulation for siRNA complexation, agarose gel electrophoresis was performed to evaluate the mass ratio of DSSP required for effective condensation LIN28B siRNA. The results showed that complete siRNA complexation was achieved when the mass ratio of DSSP to siRNA (mDSSP/msiRNA) exceeded four (Figure S3a). Quantitative analysis of siRNA band intensities further revealed that the encapsulation efficiency increased progressively with higher mass ratios, whereas the drug loading capacity gradually decreased (Figure S3b). Dynamic light scattering measurements were subsequently used to characterize the hydrodynamic diameter and zeta potential of the siRNA/DSSP nanoparticles (NPs) at various mass ratios. At a 4:1 ratio, the nanoparticles achieved a favorable hydrodynamic diameter and surface charge, suitable for downstream applications (Figure S3c–e).

**FIGURE 2:**
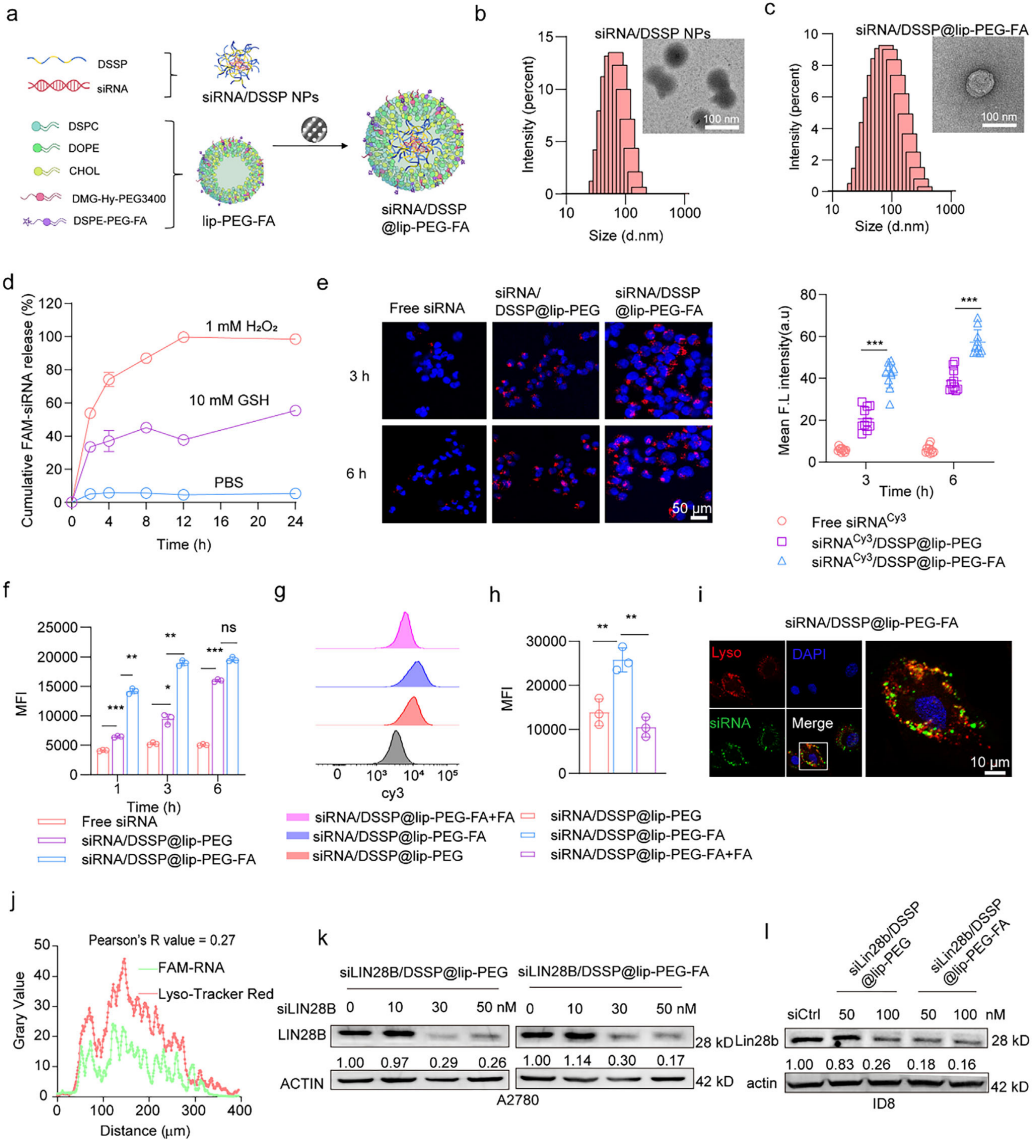
Synthesis and characterization of DSSP@lip‐PEG‐FA targeting LIN28B.(a) Schematic illustration of the synthesis process for siRNA/DSSP@lip‐PEG‐FA. Representative TEM images and size distribution profiles of siRNA/DSSP NPs (b) and siRNA/DSSP@lip‐PEG‐FA (c). (d) hydrogen peroxide (H_2_O_2_)‐ and glutathione (GSH)‐responsive siRNA release profiles under different conditions (pH 7.4, pH7.4& 1 mM H_2_O_2_, and pH 7.4 & 10 mM GSH) over 24 h. (e) Confocal microscopy images (scale bars: 50 µm) and quantitative analysis of cellular uptake efficiency for free siRNA, siRNA/DSSP@lip‐PEG, and siRNA/DSSP@lip‐PEG‐FA. (f) Flow cytometric analysis quantifying Cy3‐labeled siRNA fluorescence intensity in treated cells. (g) Flow cytometry evaluation of folate receptor (FA)‐targeting specificity. (h) Mean fluorescence intensity (MFI) comparison across different treatment groups at 6 h post‐incubation. (i) Confocal images showing intracellular distribution of siRNA/DSSP@lip‐PEG‐FA (scale bar: 10 µm). (j) Quantitative colocalization analysis performed using ImageJ software. (k) and (l) Western blot analysis of Lin28b protein expression in A2780 and ID8 ovarian cancer cells following various treatments. Data are presented as mean ± SD (*n* = 3, unpaired t‐test). *n* = independent biological replicates. *p < 0.05, **p < 0.01, ***p < 0.001; ns, not significant.

To construct targeted delivery systems, preformed liposomes were combined with siRNA/DSSP NPs and extruded 20 times through 0.45 and 0.22 µm polycarbonate membranes to generate siRNA/DSSP@lip‐PEG‐FA (Figure 2a). Compared with siRNA/DSSP NPs, the resulting siRNA/DSSP@lip‐PEG‐FA exhibited a slight increase in particle size, while maintaining uniform spherical morphology, as confirmed by transmission electron microscopy (TEM) (Figure 2b,c). Notably, TEM images revealed an additional outer lipid membrane surrounding the nanoparticle core, indicating successful liposome encapsulation.

In vitro siRNA release profiles were evaluated under oxidative and physiological conditions. The results showed that siRNA/DSSP NPs rapidly released siRNA in response to oxidative stress, whereas under phosphate‐buffered saline (PBS) conditions, less than 20% of the siRNA was released over 24 h (Figure 2d). To evaluate the in vivo stability of siRNA/DSSP@lip‐PEG‐FA, particle size and siRNA leakage were monitored in 10% FBS. As shown in Figure S3f–g, under simulated physiological conditions, the average particle size increased from ∼135 to ∼200 nm over 24 h, while siRNA leakage remained below 20% in both PBS and PBS supplemented with FBS. These results indicate that siRNA/DSSP@lip‐PEG‐FA possesses excellent redox‐responsiveness and stability, supporting its potential as a robust delivery platform for in vivo applications.

Cellular uptake of siRNA/DSSP@lip‐PEG‐FA was assessed using confocal laser scanning microscopy (CLSM) and flow cytometry. A time‐dependent increase in intracellular fluorescence was observed, with the siRNA/DSSP@lip‐PEG‐FA group exhibiting significantly higher fluorescence intensity than the non‐targeted siRNA/DSSP@lip‐PEG group (Figure 2e). In contrast, cells treated with free siRNA showed minimal fluorescence with no appreciable changes over time, indicating poor cellular internalization (Figure 2f; Figure S3h). To confirm the folate receptor‐mediated targeting capability of the DSPE‐PEG‐FA moiety, cells were preincubated with excess free folate. This pretreatment substantially reduced cellular fluorescence in the siRNA/DSSP@lip‐PEG‐FA+FA group compared with untreated cells (Figure 2g,h), demonstrating that the enhanced uptake of siRNA/DSSP@lip‐PEG‐FA is dependent on folate receptor–mediated endocytosis.

Efficient endosomal/lysosomal escape is critical for successful cytoplasmic delivery of siRNA and effective gene silencing [21, 22]. To evaluate this, the intracellular distribution of siRNA and lysosomes was examined by CLSM following a 6‐h incubation. Minimal colocalization was observed between the green fluorescence (siRNA) and red fluorescence (lysosomal marker), suggesting that a substantial portion of the siRNA had escaped from lysosomes into the cytoplasm (Figure 2i). Quantitative colocalization analysis using ImageJ revealed a Pearson's correlation coefficient of 0.27 (Figure 2j), further confirming the endosomal escape capability of siRNA/DSSP@lip‐PEG‐FA.

Next, we assessed the gene‐silencing efficacy of the LIN28B‐targeting siRNA. Treatment with LIN28B siRNA at 50 nM significantly reduced LIN28B expression at both mRNA and protein levels in A2780 cells (Figure 2k; Figure S3i). Similarly, siLin28b/DSSP@lip‐PEG‐FA treatment at 100 nM effectively downregulated Lin28b protein expression in murine ID8 ovarian cancer cells (Figure 2l). Consistent silencing effects were also observed in the human colorectal cancer cell line HCT116 (Figure S3j), demonstrating the robust and reproducible gene‐silencing capability of the nanoparticle‐delivered siRNA across multiple tumor models.

To evaluate the in vivo tumor‐targeting capability of siLin28b/DSSP@lip‐PEG‐FA, a subcutaneous colorectal cancer model was established by injecting CT26 cells into BALB/c mice. In vivo fluorescence imaging demonstrated prolonged retention and enhanced accumulation of siRNA/DSSP@lip‐PEG‐FA at the tumor site compared with the non‐targeted siRNA/DSSP@lip‐PEG formulation (Figure S4a,b). Ex vivo quantification of fluorescence in excised organs further confirmed significantly higher siRNA accumulation in tumors treated with siRNA/DSSP@lip‐PEG‐FA relative to the control group (Figure S4c). These results highlight the efficient tumor‐targeting capability of the folate‐functionalized nanoparticle system and support its potential in LIN28B‐targeted therapy.

### 2.3 siLin28b and DNA‐Damage Inhibition Cooperatively Limit MA Progression

#### 2.3.1 Synergistic Suppression of Tumor Growth by siLin28b and BMN673

Given that cancer cells with DNA repair deficiencies are particularly sensitive to PARP inhibitors [23, 24], we employed a dual‐targeting strategy combining the PARP inhibitor BMN673 with siLin28b/DSSP@lip‐PEG‐FA (siLin28b+BMN673) to investigate the therapeutic potential of simultaneously targeting LIN28B and aberrant DNA damage pathways in managing ascites. Initially, we evaluated the impact of this combination on the clonogenic capacity of A2780 and HCT116 cells. Co‐treatment with siLin28b (50 nM) and BMN673 significantly reduced colony formation at concentrations as low as 2 nM for A2780 cells and 4 nM for HCT116 cells (Figure S4d–g).

Subsequently, the in vivo antitumor efficacy of siLin28b+BMN673 was assessed. Compared with vehicle‐treated controls, the combination therapy moderately suppressed HCT116 tumor growth without inducing significant body weight loss, indicating favorable safety and tolerability (Figure S4h–k).

**FIGURE 3:**
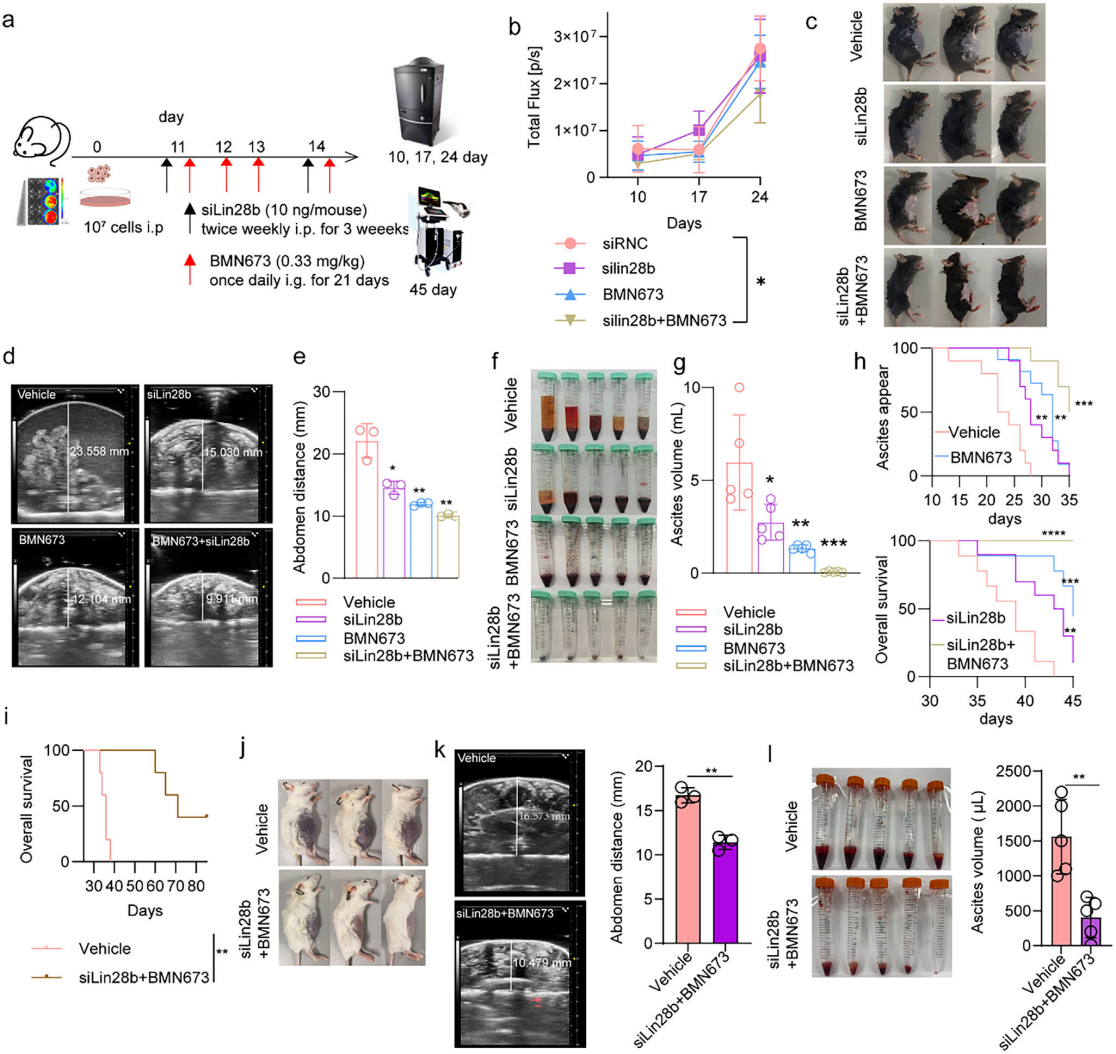
(a) Schematic illustration of the experimental design evaluating the anti‐tumor efficacy and inhibition of serous effusions following different treatments. (b) Quantification of average radiant efficiency at the ventral site over 24 days (*n* = 5, one‐way analysis of variance (ANOVA) was used for multiple comparisons among three or more groups). (c) Representative photographs of mice from each treatment group at the experimental endpoint (day 45) (*n* = 5). (d) Abdominal ultrasound images of mice after 45 days of different treatments (*n* = 3, biological replicates, unpaired t‐test). (e) Quantitative analysis of abdominal height across treatment groups (*n* = 3, biological replicates, unpaired t‐test). Representative images (f) and volumetric quantification (g) of malignant effusions collected from mice at day 45 post‐treatment (*n* = 5, unpaired t‐test). (h) Kaplan‐Meier survival curves illustrating time to onset of malignant ascites (defined by a doubling of abdominal circumference) and overall survival (*n* = 10, log‐rank test). (i) Kaplan–Meier survival curves comparing vehicle and siLin28b + BMN673 treatment groups in an independent cohort (*n* = 5 per group, log‐rank test). Representative photographs (j), ultrasound images (k), and quantification of ascites volume (l) showing reduced ascites formation after siLin28b + BMN673 combination therapy in non‐ovarian MA model (*n* = 3 or 5 per group, unpaired t‐test). *n* = independent biological replicates. All quantitative results were presented with mean ± SD. *p < 0.05, **p < 0.01, ***p < 0.001; ns, not significant.

We further evaluated this strategy in a preclinical ovarian cancer model. Bioluminescence imaging revealed that siLin28b+BMN673 treatment modestly reduced tumor burden in ID8‐Luciferase‐bearing mice compared with vehicle‐treated controls, whereas monotherapy with either agent had no significant effect (Figure 3a,b; Figure S4l). Immunohistochemical analyses confirmed the enhanced antitumor effect of the combination therapy, showing a marked reduction in Ki67‐positive proliferating cells and a pronounced increase in TUNEL‐positive apoptotic cells (Figure S4m,n).

#### 2.3.2 Synergistic suppression of MA by siLin28b and BMN673

In contrast to their limited effect on tumor burden, monotherapy with siLin28b or BMN673 moderately reduced malignant ascites volume, whereas the combination therapy produced the most pronounced effect. By day 45, the abdominal girth of vehicle‐treated mice nearly doubled compared to mice receiving siLin28b+BMN673, indicating substantial peritoneal fluid accumulation (Figure 3c; Figure S5a). Ultrasound imaging further confirmed that combination treatment markedly decreased abdominal fluid height, with treated mice exhibiting an average abdominal height of <10 mm vs. >20 mm in controls (Figure 3d,e; Figure S5b). Notably, the combination therapy yielded the lowest ascites volume, whereas other treatment groups developed ascites ranging from 1 to 10 mL (Figure 3f,g).

Moreover, the onset of MA was markedly delayed in the combination treatment group. Whereas MA first appeared on day 13 in vehicle‐treated mice, ascites development was postponed to day 24 and day 22 in the siLin28b and BMN673 monotherapy groups, respectively. In contrast, mice receiving the combined siLin28b+BMN673 treatment did not develop ascites until day 28 (Figure 3h). Consequently, this group exhibited the longest ascites‐free survival and overall survival among all treatment groups. Long‐term survival analysis further demonstrated that 40% of mice remained healthy at day 85 following siLin28b+BMN673 treatment (Figure 3i).

#### 2.3.3 siLin28b plus BMN673 Suppresses MA Accumulation in Non‐Ovarian MA Models

To evaluate the therapeutic efficacy of the siLin28b+BMN673 combination in a non‐ovarian malignant ascites model, we established a CT26‐derived ascites model. As shown in Figure S5c, CT26 cells expressed Lin28b protein at levels comparable to those in ID8 cells, and both qPCR and western blot analyses confirmed that 50 nM siLin28b effectively suppressed Lin28b expression (Figure S5d,e). The results demonstrated that the combination treatment remained highly effective in inhibiting ascites progression. Specifically, siLin28b combined with BMN673 markedly reduced abdominal girth (Figure 3j,k), and abdominal height was significantly lower than in the vehicle group. Consistently, the ascites volume was also substantially reduced in the combination‐treated mice (Figure 3l).

### 2.4 siLin28b Plus BMN673 Remodels the Ascites Microenvironment by Modulating M2 Macrophages and Neutrophil Subtypes

**FIGURE 4:**
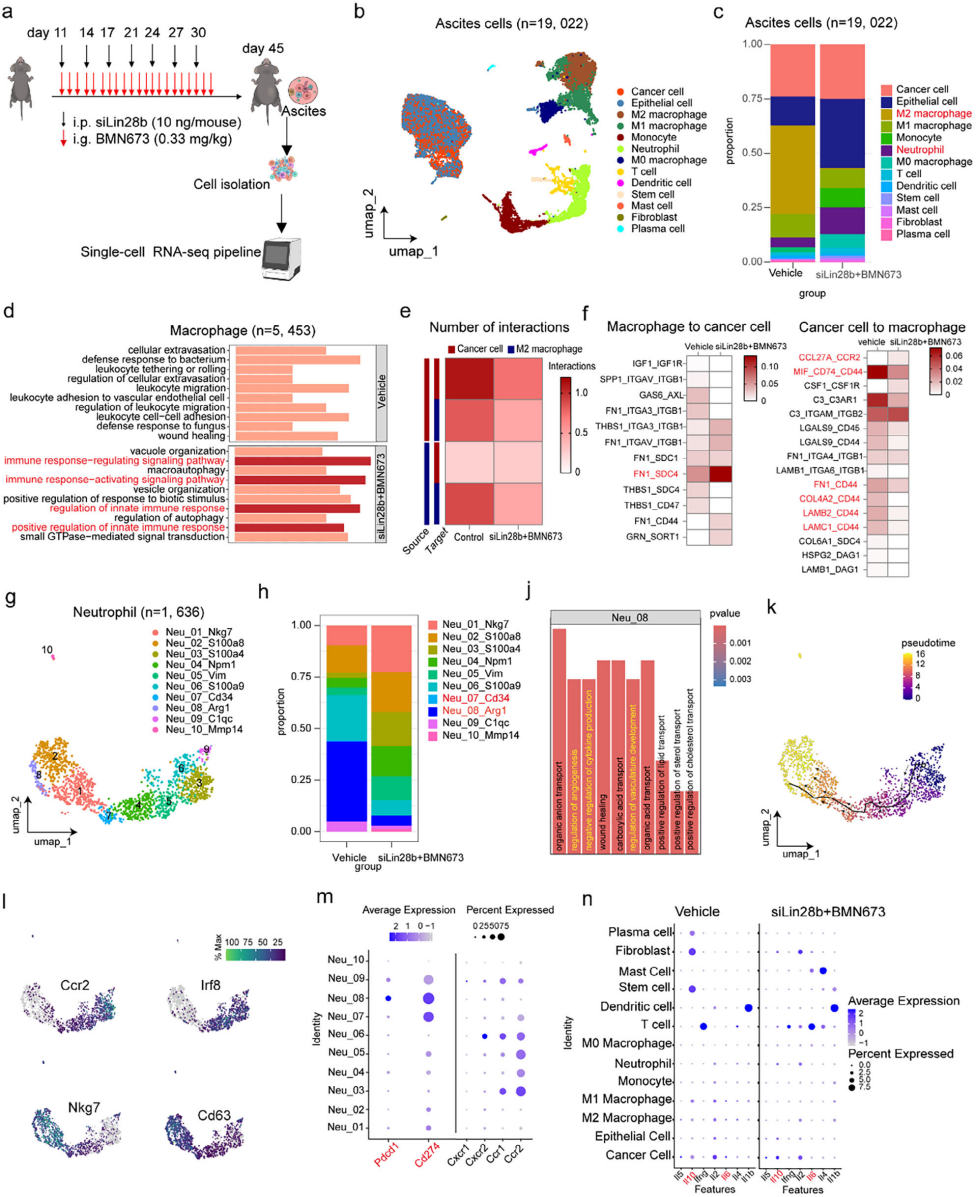
siLin28b plus BMN673 remodels the ascites microenvironment. (a) Establishment of the preclinical OC model. (b) UMAP plots of all MA cells (*n* = 19, 022). (c) Proportion histograms of all cell‐types in the vehicle and siLin28b+BMN673 groups (*n* = 5). (d) GO‐BP enrichment analysis of macrophage subtypes (*n* = 5, 453). (e) Heatmap of Interactions (calculated using the “CellChat” R package) between cancer cells and M2 macrophages in vehicle and siLin28b+BMN673 groups. (f) Heatmap showing receptor‐legends interactions between cancer cells and macrophages in vehicle and siLin28b+BMN673 groups. (g) UMAP plots of neutrophil subtypes (*n* = 1, 636) within malignant serous effusions. (h) Proportional histograms of neutrophil subtypes across vehicle and siLin28b+BMN673 groups. (i) GO‐BP enrichment analysis of the Neu‐08 neutrophil subpopulation. (j) Pseudotime trajectory analysis of neutrophil subtype evolution. (k) UMAP plots depicting the expression patterns of ccr2, irf8, nkg7, and cd63 across neutrophil subtypes. (l) Dot plots showing the expression of pdcd1, cd274, cxcr1, cxcr2, ccr1, ccr2 in neutrophil subsets. (m) Dot plots displaying immune stimulating factor expression across all cell types.

Previous studies have demonstrated that alterations in the MA microenvironment serve as a critical indicator of MA progression [19]. To investigate how treatment modulates the immune cell composition within the MA microenvironment, we performed scRNA‐seq analysis (Figure 4a). A total of 19022 cells derived from the ID8 mouse model were analyzed and clustered into distinct cell populations (Figure 4b). To accurately delineate malignant cells in both siLin28b+BMN673 and control groups, we applied the Copy number Karyotyping of Aneuploid Tumors (CopyKAT) algorithm, which infers genomic copy number variations from scRNA‐seq data [25]. By integrating the expression profiles of Carcinoembryonic Antigen‐Related Cell Adhesion Molecule (CEACAM) family genes, we further refined tumor cell annotation. As shown in Figure S6a, CEACAM genes were highly expressed in aneuploid cells identified by CopyKAT, while exhibiting low expression in diploid cells. Notably, LIN28B expression was substantially upregulated in the cancer cell cluster but was markedly attenuated following combination treatment with siLin28b and BMN673 (Figure S6b). Marker genes for each cell cluster are provided in Figure S6c.

Strikingly, the proportion of M2‐like macrophages was significantly reduced after combination therapy (Figure 4c), consistent with previous reports demonstrating that macrophage accumulation in MA contributes to tumor progression and immune evasion [16].

Gene Ontology Biological Process (GO‐BP) analysis of macrophages revealed significant enrichment of immune‐related pathways following combination treatment, including “immune response,” “regulation of innate immune response,” and “positive regulation of innate immune response” (Figure 4d). These results indicate an enhanced activation of innate immune programs in macrophages upon siLin28b+BMN673 treatment.

Furthermore, cell–cell interaction analysis demonstrated a marked reduction in crosstalk between M2‐like macrophages and cancer cells after combination therapy (Figure 4e). Consistently, CellChat‐based cell–cell communication analysis identified a substantial decrease in CD44‐associated ligand–receptor interactions between tumor cells and macrophages, suggesting disruption of macrophage‐mediated immunosuppressive signaling pathways (Figure 4f). Collectively, these findings demonstrate that siLin28b+BMN673 not only reshapes macrophage polarization but also attenuates their pro‐tumoral interactions with cancer cells, thereby contributing to the reprogramming of the malignant ascites immune microenvironment.

Our analysis further revealed pronounced alterations in the neutrophil population following treatment (Figure 4c). To characterize neutrophil heterogeneity in greater detail, we analyzed 1636 neutrophils and identified ten distinct subpopulations based on their unique marker gene expression profiles (Figure 4g). Notably, the Neu‐07‐Cd34 and Neu‐08‐Arg1 subtypes were significantly depleted following combination therapy with siLin28b and BMN673 (Figure 4h). GO‐BP enrichment analysis indicated that the Neu‐08‐Arg1 subtype is involved in the negative regulation of cytokine production and vasculature development, implicating this population in ascites formation and therapeutic resistance (Figure 4l). Consistent with this notion, pseudotime trajectory analysis suggested that both Neu‐07‐Cd34 and Neu‐08‐Arg1 subtypes likely originate from the Neu‐09‐C1qc cluster (Figure 4j), suggesting a shared developmental trajectory. Further stratification revealed two major neutrophil phenotypic programs: one characterized by high expression of *Ccr2* and *Irf8*, and another marked by elevated levels of *Nkg7* and *Cd63*, genes previously associated with tumor invasion and metastatic potential (Figure 4k; Figure S6d). Notably, neutrophil subsets associated with poor prognosis‐including Neu‐07‐Cd34, Neu‐08‐Arg1, Neu‐01‐Nkg7, and Neu‐02‐S100a8‐exhibited high expression of immune exhaustion markers *Pdcd1* and *Cd274*. In contrast, key chemokines such as *Ccr1*, *Ccr2*, *Cxcr1*, and *Cxcr2* were expressed at significantly lower levels in these subsets compared with the Neu‐09‐C1qc cluster and related populations (Figure 4m).

In parallel, cytokine expression analysis revealed that siLin28b+BMN673 treatment reduced the levels of IL‐6 and IL‐10 in macrophages and cancer cells, while concomitantly increasing their expression in T cells (Figure 4n). These findings suggest that the therapeutic efficacy of the combination treatment arises from coordinated, multicellular remodeling of the malignant ascites microenvironment rather than effects on a single cell type.

Collectively, these results underscore the multifaceted role of neutrophils in the MA microenvironment and identify immune‐suppressive neutrophil subtypes (Neu‐07‐Cd34, Neu‐08‐Arg1, Neu‐01‐Nkg7, and Neu‐02‐S100a8) as critical contributors to disease progression. Targeting these pathological neutrophil states may therefore represent an effective strategy to enhance MA management.

To determine which immune cell population plays a major role, we performed targeted depletion based on our single‐cell analysis. By combining anti‐CD8 treatment with siLin28b+BMN673 therapy, we found that depletion of CD8^+^ T cells did not affect the inhibitory effect of the combination treatment on ascites formation (Figure S7a). In contrast, selective depletion of neutrophils significantly suppressed ascites and markedly reduced abdominal height, whereas depletion of macrophages had no such effect (Figure S7b). Consistently, neutrophil depletion led to a survival benefit compared with the vehicle group, while macrophage depletion provided no improvement (Figure S7c). Taken together, these results indicate that neutrophils play a critical role in regulating ascites formation.

### 2.5 The siLin28b Plus BMN673 Combination Inhibits the Deterioration of Vascular Permeability

**FIGURE 5:**
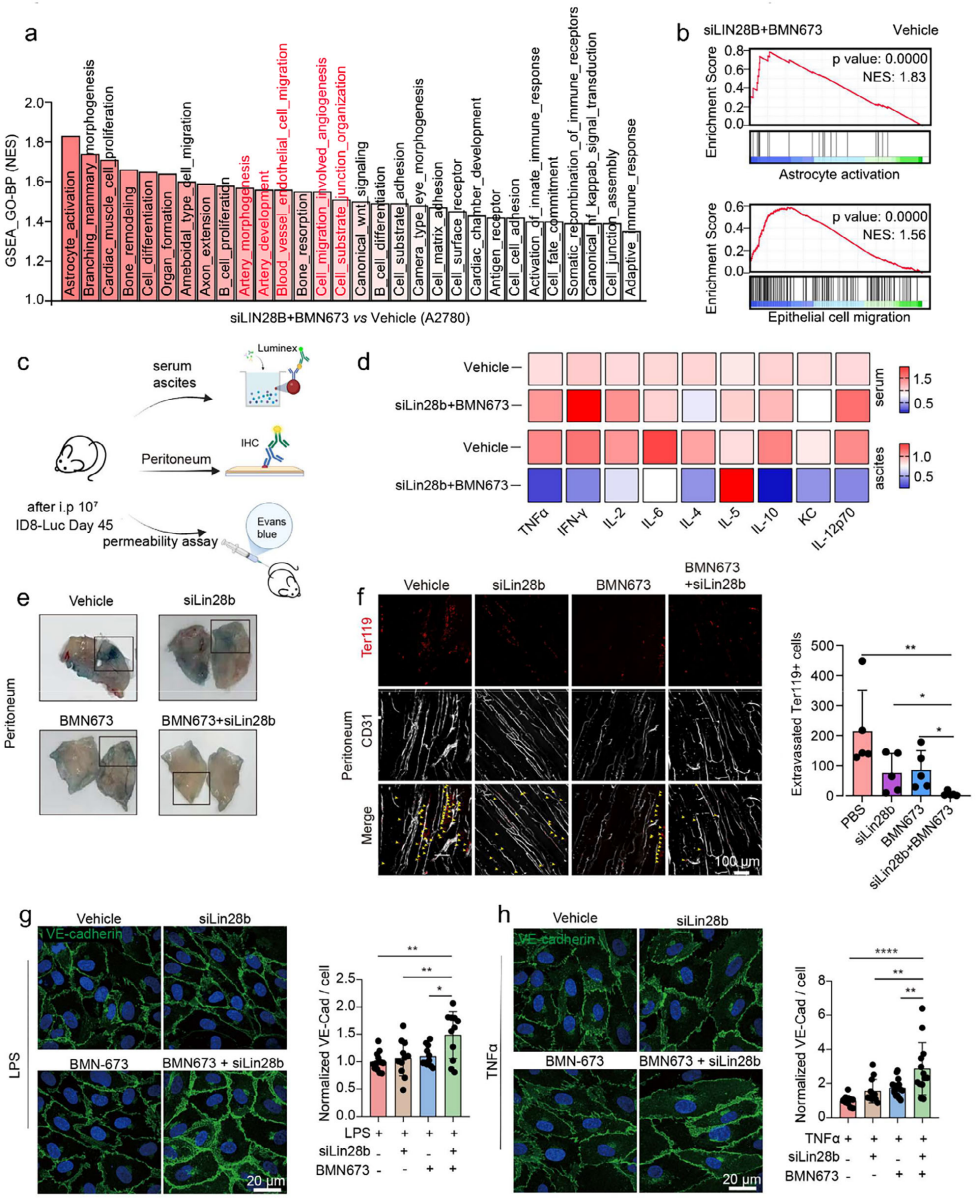
The siLin28b plus BMN673 combination inhibits the deterioration of vascular permeability. (a) GO‐BP enrichment analysis identifying the top 30 pathways significantly enriched between siLIN28B+BMN673 and PBS‐treated A2780 cells. (b) Enrichment of astrocyte activation and endothelial cell migration pathways in A2780 cells following siLin28b and BMN673 treatment. (c) Schematic workflow illustrating the mechanistic investigation of ascites suppression (created at BioRender.com). (d) Heatmap of cytokine profiles in MA and blood samples detected by Luminex xMAP technology. (e) Representative images of peritoneal Evans blue permeability assays. (f) Immunofluorescence staining of peritoneal tissues across different treatment groups (scale bar: 100 µm). (g) Representative images depicting the effects of siLin28b, BMN673, and their combination on adherent junctions in LPS stimulated HUVECs, assessed by VE‐Cadherin staining. (h) Representative images illustrating the effects of siLin28b, BMN673, and their combination on adherent junctions in TNFα‐challenged HUVECs. All quantitative results are presented as mean ± SD, *n* = 3, unpaired t‐test. *n* = independent biological replicates. *p < 0.05, **p < 0.01, ***p < 0.001.

To investigate how siLin28b+BMN673 modulate MA, we analyzed RNA‐seq data from A2780 cells subjected to different treatments. GO‐BP pathway analysis, ranked by p‐value and normalized enrichment score, revealed significant upregulation of pathways related to endothelial cell migration, and junctional organization in the siLin28b+BMN673 group compared with the vehicle group (Figure 5a,b), suggesting the involvement of altered vascular function. We further collected serum, ascites, and peritoneal fluid from tumor‐bearing mice and quantified cytokine levels using Luminex assays (Figure 5c). While serum cytokine levels remained largely unchanged, ascitic fluid exhibited marked alterations post‐treatment. Specifically, combinational therapy significantly reduced the secretion of pro‐inflammatory cytokines, including TNF‐α, IL‐6, and IL‐10, in the MA microenvironment compared to vehicle treatment (Figure 5d). Notably, all mice in the combination treatment group survived until day 45, contrasting with those receiving single‐agent therapies. ELISA validation confirmed reduced levels of IL‐6, IL‐10, and GM‐CSF in ascitic fluid, aligning with our observation that combinational therapy effectively inhibits MA accumulation (Figure S8a). Meanwhile, compared with the vehicle group, vascular endothelial growth factor (VEGF)‐A levels were significantly reduced in the siLin28b plus BMN673 group, whereas bFGF levels remained unchanged (Figure S8b).

Next, we assessed vascular permeability using Evans blue dye. Mice treated with siLin28b+BMN673 showed minimal Evans blue retention in the peritoneum, indicating markedly reduced vascular leakage compared to the vehicle‐treated controls (Figure 5e). Immunostaining with Ter119 and CD31 revealed extensive erythrocyte extravasation in vehicle‐treated mice. In contrast, monotherapy with either siLin28b or BMN673 monotherapy reduced leaked erythrocytes, while their combination achieved the most significant reduction, reflecting enhanced vascular integrity (Figure 5f).

To assess whether depletion of specific cell populations improves vascular integrity, we collected peritoneal tissues from the relevant experimental groups for immunohistochemical analysis. As shown in Figure S9a, combining anti‐CD8 treatment did not affect the vascular improvements induced by siLin28b+BMN673. In contrast, selective depletion of neutrophils markedly reduced erythrocyte extravasation compared with the vehicle group, whereas macrophage depletion had no effect on red blood cell leakage (Figure S9b,c). Taken together, these results indicate that neutrophils play a critical role in regulating vascular integrity.

To investigate whether this improved vascular integrity was directly caused by the combination treatment, we treated confluent HUVEC monolayers with siLin28b, BMN673, or both in the presence of TNF‐α or LPS. As shown in Figure S9d, LPS, TNF‐α, and IL‐6 all induced upregulation of Lin28b expression in HUVECs. The combination treatment effectively restored disrupted endothelial junctions to continuous, reticular‐like patterns, indicating improved endothelial monolayer integrity and tight junction formation (Figure 5g,h). Functionally, recombinant IL‐6 and TNF‐α significantly increased endothelial monolayer permeability, as evidenced by enhanced FITC‐dextran leakage. In contrast, neutralizing antibodies against IL‐6 or TNF‐α effectively reversed these cytokine‐induced permeability changes, restoring endothelial barrier integrity (Figure S9e). These results highlight enhanced vascular integrity as a critical mechanism underlying the therapeutic efficacy of siLin28b+BMN673 in managing MA.

Biosafety is a critical consideration for the clinical application of any therapeutic regimen. Subsequently, we evaluated the biosafety profiles of siLin28b+BMN673 in tumor‐free mice. Major organs including the heart, liver, spleen, lung, and kidney were collected and subjected to H&E staining. The histopathological analysis revealed no significant differences between the groups treated with siLin28b+BMN673 or vehicle, with nuclear and cellular morphologies remaining unchanged post‐treatment (Figure S10a). Consistently, a comprehensive blood analysis assessing six key toxicity parameters demonstrated comparable levels between the siLin28b+BMN673 and vehicle groups (Figure S10b), indicating no discernible impact on hepatic or renal function. In addition, cTnI staining of myocardial tissue revealed no detectable differences between the treatment and vehicle groups (Figure S10c). TUNEL staining of major organs similarly showed no signs of cardiac or tissue damage (Figure S10d). We further assessed serum IL‐6 and anti‐PEG antibody levels, and neither exhibited abnormal elevation, thereby excluding the risk of a cytokine storm. Consistently, no significant increases were observed compared with the vehicle group (Figure S10e). Collectively, these findings demonstrate that siLin28b+BMN673 construct is well‐tolerated and does not induce systemic toxicities, supporting its potential for clinical use.

## 3 Discussion

**FIGURE 6:**
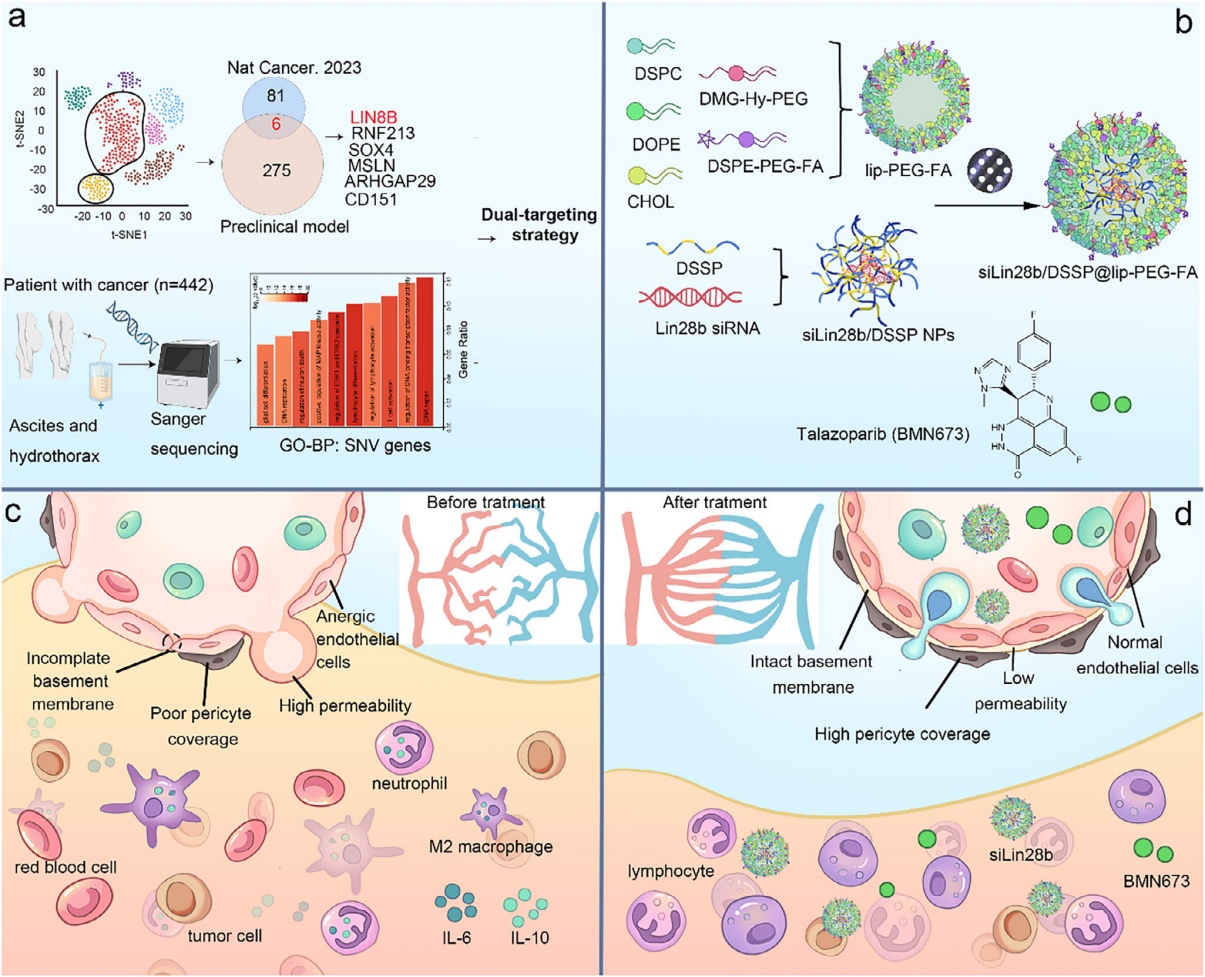
Working model. The combination of siLin28b/DSSP@lip‐PEG‐FA and BMN673 effectively suppresses the accumulation of malignant ascites. (a) This study integrates three data sources to propose a novel dual‐targeting therapeutic strategy: scRNA‐seq analysis of ascites from a preclinical OC model, scRNA‐seq of ascites from 32 OC patients (*n* = 13), and gene mutation profiling of polyserous effusions from cancer patients (*n* = 442). (b) The synthesis process of siLin28b/DSSP@lip‐PEG‐FA involved compressing siLin28b using DSSP, followed by targeted modification with DSPE‐PEG‐FA to enhance the in vivo targeting and delivery efficiency of siLin28b, as well as the structure of the PARP1 inhibitor BMN673. Compared to the vehicle group (c), the combination therapy (d) improved vascular integrity and reduced vascular permeability and significantly inhibited the infiltration of M2‐polarized tumor‐associated macrophages (TAMs), along with decreased secretion of pro‐tumorigenic cytokines TNF‐α, IL‐6, and IL‐10.

In this study, we uncovered an unexpected role for LIN28B and PARP in MA and developed a novel therapeutic strategy that combines tumor‐targeted LIN28B siRNA nanoparticles with the PARP inhibitor BMN673 to manage MA accumulation (Figure 6). LIN28B, initially identified in hepatocellular carcinoma [26], is an evolutionarily conserved RNA‐binding protein that regulates diverse biological processes, including development and cancer [27, 28]. Mechanistically, LIN28B is known to sequester pri‐let‐7 transcripts in the nucleolus, thereby preventing their maturation [29]. In addition to this canonical function, our previous studies demonstrated let‐7‐independent mechanisms by which LIN28B modulates the AKT2/FOXO3A/BIM axis [30], and translationally represses p53 to promote tumor growth [31]. Given its selective reactivation in malignant tissues relative to normal tissues [32, 33], LIN28B presents an ideal target for cancer‐specific therapies, consistent with the minimal systemic toxicity observed in our treatment regimen.

Importantly, the siRNA sequence used in our study was specifically designed to selectively target the LIN28B transcript rather than the LIN28A transcript. Although LIN28A and LIN28B are homologous RNA‐binding proteins, they are not functionally redundant; instead, they operate in distinct cellular compartments and biological contexts. Notably, prior studies have demonstrated that LIN28A does not meaningfully compensate for LIN28B loss in malignant ascites models [29, 34]. These findings suggest that LIN28A plays a minimal role in our study, and LIN28B is the dominant regulator of the phenotypes in our model. However, it still merits further investigation of co‐targeting both LIN28B and A in cancer models where they are co‐expressed to test the compensatory effects.

Mutations or loss of function in DNA repair genes can impair homologous recombination and drive tumorigenesis [35, 36]. Such defects often create cancer‐specific vulnerabilities that can be exploited through synthetic lethality [37]. Poly (ADP‐ribose) polymerase inhibitors (PARPis) have attracted significant attention in cancer therapy, primarily as agents targeting impaired DNA repair pathways via synthetic lethality [38, 39]. PARPis, including Olaparib and Niraparib, have been explored as maintenance therapies [40, 41, 42, 43, 44], and are associated with both tumor cell control and immune system activation [45, 46, 47, 48]. Despite the implication of PARP in vascular disorders [49, 50], the effects of PARPis in this context remain poorly understood. Our findings reveal a novel role for PARP beyond its canonical functions in cancer, highlighting its regulation of vascular integrity.

Beyond standard therapies, several experimental approaches are under investigation, including oncolytic adenovirus H101 [51], immunotherapies [52, 53], and targeted therapies [54, 55, 56]. Among these, VEGF inhibitors have garnered substantial interest. MA is thought to arise mainly from impaired vascular integrity, which is associated with excessive VEGF secretion by cancer and stromal cells [57, 58]. VEGF inhibition (VEGFi) has been tested as a potential MA treatment, with some success in reducing MA accumulation [59]. However, VEGFi is associated with severe toxicities, including gastrointestinal complications such as perforation and obstruction, and in some cases, fatal sepsis [60]. These adverse effects substantially limit the clinical application of VEGFi in MA management. Given the cancer‐specific expression pattern of LIN28B and the targeted nature of PARPis, our study proposes a safer combinational approach for effective MA management.

This study has several limitations. First, the precise molecular mechanisms underlying MA management require further elucidation. Although we demonstrate that combining PARPis with LIN28B siRNA strengthens epithelial tight junctions, the exact mechanisms remain unclear. We speculate that multiple pathways, involving both cancer‐specific and non‐cancer‐specific effects, synergistically enhance therapeutic efficacy. Second, scRNA‐seq of ascites cells revealed LIN28B reactivation in both cancer and immune cells. The impact of combination therapy on immune cell function, and the role of immune cell LIN28B in regulating treatment efficacy‐including vascular permeability and cell‐cell tight junctions‐warrant further investigation. Third, while our approach does not alter the PARPi (BMN673) treatment regimen and is readily translatable to clinical settings, good manufacturing practice‐compliant humanization of the LIN28B siRNA‐incorporating nanoparticle is necessary for future clinical trials of this combination therapy.

## 4 Experimental and Methods

### 4.1 Materials

#### 4.1.1 Patient Samples

Written consent was obtained from participants permitting the utilization of discarded serous effusion samples for research purposes, in accordance with an Institutional Review Board‐approved protocol at Tongji Hospital, Tongji Medical College, Huazhong University of Science and Technology (*Approval number: TJ‐IRB202407052*). A total of 442 patients were prospectively recruited between July 1, 2024 and March 31, 2025. All clinicopathologic characteristics were collected prospectively in a centralized database, an unidentified version of which is made available in Table S1.

#### 4.1.2 Plasmids and Antibodies

For LIN28B knockdown, siLIN28B was transfected into A2780, ID8, and HCT116 cells using siRNA/DSSP@lip‐PEG or siRNA/DSSP@lip‐PEG‐FA. The target guide sequences used are listed:

siLIN28B Human 5′‐CCUCCACGAAGUCAUCUAUtt‐3′;
5′‐AUAGAUGACUUCGUGGAGGtt‐3′;
shlin28b mouse 5′‐GGAUGUAUUUGUACACCAAtt‐3′;
5′‐UUGGUGUACAAAUACAUCCtt‐3′.

The following antibodies were used: anti‐human LIN28B (Cell Signaling Technology, 4196S, 1:1000), anti‐mouse Lin28b (Proteintech, 11724‐1‐AP, 1:500), anti‐β‐actin (ABclonal, AC026, 1:10000), StarBright Blue 520 goat anti‐mouse IgG (Bio‐Rad, 12005867, 1:10000), Alexa Fluor Plus 800 goat anti‐rabbit IgG (H + L) (Invitrogen, A32735, 1:10000), Anti‐Mouse CD8a‐Purified In vivo (C375‐25 mg), Anti‐Mouse NK1.1‐Purified In vivo (N123‐25 mg), Human/Primate IL‐6 Mab(Clone 6708, MAB206‐SP, R&D), Human TNF‐alpha Mab(Clone28401, MAB610‐SP, R&D), anti‐CD31 antibody (Thermo Fisher Scientific, MA3105, 1:200), and anti‐TER‐119 antibody (BD Biosciences, 557915, 1:100).

#### 4.1.3 Chemical Compounds

2, 2′‐Thiobis(ethylamine) (Jiangsu Aikang Biomedical R&D Co., Ltd., catalog number: 871‐76‐1), acryloyl chloride (Tianjin Xiensi Biochemical Technology Co., Ltd., catalog number: 814‐68‐6), spermine (Tianjin Xiensi Biochemical Technology Co., Ltd., catalog number: 71‐44‐3), N,N'‐Methylenebisacrylamide (Tianjin Xiensi Biochemical Technology Co., Ltd., catalog number: 110‐26‐9), N,N‐Dimethylethylenediamine (Tianjin Xiensi Biochemical Technology Co., Ltd., catalog number: 108‐00‐9), cystamine dihydrochloride (Tianjin Xiensi Biochemical Technology Co., Ltd., catalog number: 56‐17‐7), folate‐conjugated polyethylene glycol phospholipid (DSPE‐PEG‐FA) (Aladdin, catalog number: B2224303), DMG‐Hy‐PEG3400 (purchased from Ruixi Biological, catalog number: R‐S0011), dioleoylphosphatidylethanolamine (DOPE) (Ruixi Biological, catalog number: 4004‐05‐1), distearoylphosphatidylethanolamine (DSPE) (Ruixi Biological, catalog number: 1069‐79‐0), and cholesterol (Tian'jin Xiensi Biochemical Technology Co., Ltd., catalog number: 57‐88‐5), Recombinant Human IL‐6(HEK293‐expressed) Protein (7270‐1L‐025, R&D), Clodronate Liposomes(From Vrije Universiteit Amsterdam, 40337ES10, Yeasen), Mouse anti‐PEG (Polyethylene glycol) IgG ELISA kit (EM2149, Fine Test, Wuhan Fine Biotech Co., Ltd.), Mouse bFGF ELISA Kit (Solarbio, SEKM‐0081), Mouse VEGF‐A ELISA Kit (bioswamp, MU30660),Mouse IL‐6 ELISA Kit (Multisciences, EK206), Mouse IL‐β ELISA Kit (Multisciences, 410 EK201B), Mouse IL‐1α ELISA Kit (Multisciences, EK201B), Mouse IL‐10 ELISA Kit (Multisciences, EK210), and Mouse GM‐CSF ELISA Kit (Multisciences, EK263).

### 4.2 Bulk and Single‐Cell Transcriptomic Analyses

#### 4.2.1 DNA Extraction and Library Preparation

DNA extraction was performed using the QIAamp DNA FFPE Tissue Kit from Qiagen (Valencia, California), according to the manufacturer's instructions. The DNA concentration of MSE samples was measured using the Qubit 2.0 Fluorometer along with the Qubit dsDNA Assay Kit (Life Technologies, Carlsbad, California). A minimum of 50 ng of DNA was required for NGS library construction [61]. DNA fragmentation was achieved using the Covaris M220 Focused‐ultrasonicator (Covaris, Woburn, MA), followed by end repair, phosphorylation, dA‐tailing, and adapter ligation to construct the sequencing libraries. The resulting DNA libraries were purified using Agencourt AMPure beads (Beckman Coulter, Fullerton, CA).

#### 4.2.2 Mutational Signature Analysis

We estimated contributions of COSMIC 50 mutational signatures to an observed mutational spectrum in each sample using the deconstructSigs package (v 1.8.0) in R. Exome regions were defined by the Agilent SureSelect V6 target region. We conducted de novo mutational signatures finding by identifySignatures function of SomaticSignatures R package (v 2.34.0). Only somatic mutations in exome regions were considered. Mutalisk (a web‐based somatic MUTation AnaLysIS toolKit; Nucleic Acids Research, 2018. http://mutalisk.org/) was used to analyze somatic local hypermutation.

#### 4.2.3 CNV Analysis

The InferCNV R package (v 1.6.0) was used to call copy number changes from OC patients (*n* = 442). The UMI counts were used as input to infer copy number changes. Endothelial cells and fibroblasts were considered as control samples. An inferCNV object was created using the *CreateInfercnvObject* function with raw UMI counts and hg38 genomic annotations as input. Run function parameters were set as follows: cutoff = 0.1, cluster_by_groups = TRUE, denoise = TRUE, HMM = TRUE, num_threads = 4. The matrix generated from InferCNV containing the initial CNVs for each region were used to calculate the CNV scores of total cell types. For each sample, gene expression of cells was re‐standardized and values were limited as −1–1. The CNV score of each cell was calculated as quadratic sum of CNV_region_.

#### 4.2.4 Single‐Cell RNA‐seq Data Processing

In‐house FASTQ files generated from 10×Genomics were aligned and quantified using Cell Ranger software (Version 6.1.2) with default settings. The output of the cell ranger and count matrix were read using the Read10× function from the *Seurat* package (Version 4.0.4). The merge function was used to integrate individual objects into an aggregate object, and the *RenameCells* function was used to ensure that all cell labels were unique. In total, 28 of 378 cells from GC tissues of different patients were pooled. Quality control was applied to cells based on several criteria. Briefly, cells with <200 detected genes and >20% mitochondrial content were excluded. Cells with >6000 detected genes were eliminated to exclude possible doublets. After filtering, 19, 022 high‐quality cells were preserved for subsequent analyses. A global‐scaling normalization method (“LogNormalize”) was employed to ensure that the total gene expression in each cell was equal, and the scale factor was set to 10 000. The top 2000 variably expressed genes were returned for downstream analysis using the *FindVariableFeatures* function. The *RunFastMNN* function in *SeuratWrappers* package (version 0.3.0) was used for sample batch correction. Clustering analysis was performed based on edge weights between any two cells, and a shared nearest‐neighbor graph was produced using the Louvain algorithm, implanted in the *FindNeighbors* and *FindClusters* functions. Identified clusters were visualized using the UMAP method. A similar procedure was applied for neutrophil sub‐clustering analysis, including normalization, variably expressed feature selection, dimension reduction, batch correction with *RunFastMNN*, and clustering identification.

#### 4.2.5 Gene Differential Expression Analysis

DEGs were detected using DESeq2 (v 1.26.0) based on absolute log2 transformed fold‐change values > 2 and adjusted values of *p* < 0.05 after applying the Benjamini–Hochberg correction. All R packages were run in version 4.0.3. The gene set enrichment analysis (GSEA) (v 4.2.3) was used to explore the whole‐transcriptome dataset.

#### 4.2.6 Pseudotime Trajectory Analysis

To construct single cell pseudotime trajectories and to identify gene expression changes as the cells undergo transition, the Monocle 2 (v 2.18.0) algorithm was applied to the cell subtypes. Top 2000 most variable genes with expressions in ≥ 10 cells and q‐value < 0.01 were used for trajectory inference. Cells were ordered along the trajectory, and their trajectory was visualized in the reduced dimensional space. Significantly changed genes along the pseudotime trajectory were identified using the differential Gene Test function of Monocle 2 and showed by branched‐heatmap images and/or scatter plot images.

#### 4.2.7 Cell Communication

Cell communication analysis was performed using the R package *CellChat* (v1.0.0) with default parameters. The CellChatDB human was used for analysis. In computing the communication probability, 10% truncated mean was used, and the communication was filtered out if there are less than 10 cells in certain cell groups. Cell types of each group were normalized together, and each cluster was extracted and analyzed and compared in parallel.

#### 4.2.8 RNA‐seq

RNA‐seq was performed by Genewiz (South Plainfield, New Jersey, USA) using chloroform extraction and isopropanol precipitation, following previously described protocols. RNA‐seq libraries were prepared using the SMART‐Seq kit (CloneTech) and subsequently fragmented with the Nextera XT kit (Illumina). Sequencing was conducted on an Illumina HiSeq 2500 (Illumina), generating either paired‐end reads of either 100 or 150 base pairs or single‐end reads of 100 base pairs. RNA‐seq reads were aligned to the human genome (GRCh38) using STAR (v2.7.7a) and quantified with RSEM (v1.3.3), based on GENCODE version 36 annotations. Raw counts of transcripts sharing the same gene symbol were aggregated. Non‐protein‐coding genes, pseudogenes, predicted genes, genes with less than 1 FPKM in at least one sample within a sample group (tissue/disease group), and genes with low variance were excluded from downstream analysis. Batch effects were corrected using ComBat (sva v3.38.087). Differential expression analysis was performed using *limma* (v3.46.0), identifying genes with a p‐value |log_2_FC| > 1.5 and a p‐value < 0.05 considered significantly differentially expressed between A2780 treated with siLIN28B + BMN673 and the Vehicle group (*n* = 3). Pathway analyses (GSEA) were conducted using the *clusterProfiler* R package (v3.18.1).

### 4.3 SNVs and CNVs Analyses

#### 4.3.1 Data Preprocessing

Raw SNV/CNVs sequencing reads (FASTQ files) from cancer patients (*n* = 442) undergo a standardized preprocessing pipeline prior to variant calling. FastQC and Trimmomatic are used to assess read quality. Adapter sequences and low‐quality bases (typically Phred score <20) are trimmed from read ends. Reads are aligned to a reference genome (e.g., GRCh38) using aligners such as Bowtie2, resulting in SAM/BAM files. The aligned data is then processed using SAMtools/Picard to sort alignments and mark PCR duplicates to minimize artifacts.

#### 4.3.2 Statistical Methods and Calling

For SNV calling, the Genome Analysis Toolkit Best Practices are followed. Metrics include Quality by Depth (QD < 2.0), strand bias (Fisher Strand, FS > 60.0), RMS Mapping Quality (MQ < 40.0), and read position bias (ReadPosRankSum < −8.0). This involves local realignment around indels and BQSR to correct systematic base quality score errors. For CNV detection via depth‐of‐coverage methods, additional normalization steps are critical. This includes correcting for genomic regions with extreme GC content, mappability biases, and sample‐specific library size differences. Segment‐wise log2 ratio standard deviation or confidence score (CNVkit quality score > 0.5). Typically, > 1 kb for targeted sequencing and > 10–50 kb for whole‐genome sequencing to exclude small, noisy calls. Log2 ratio thresholds define copy states (deletion: log2 ratio < −0.4, duplication: log2 ratio > 0.2).

The normalized coverage is assumed to follow a Poisson or negative binomial distribution to statistical modeling. Segmentation algorithms identify changepoints where the mean coverage significantly deviates from the expected baseline (ploidy = 2). B‐allele frequency (BAF) from SNP arrays or sequencing is integrated to improve precision in determining allelic‐specific copy number states.

### 4.4 Synthesis of DSSP

2,2′‐Thiobis(ethylamine) (0.05 mol) was dissolved in dichloromethane (DCM) in a three‐necked flask and cooled to 0–5 °C in an ice‐water bath. Aqueous NaOH solution (0.4 mol, 20 mL) and acryloyl chloride (0.2 mol, 10 mL) in DCM were simultaneously added dropwise under vigorous stirring. After stirring in the ice‐water bath for 1.5 h, the reaction mixture gradually warmed to room temperature and allowed to react overnight. The mixture was then extracted with DCM, concentrated under reduced pressure, and washed repeatedly with water to yield TCBA.

Subsequently, spermine was dissolved in 30 mL of a 10% (v/v) methanol/water solution in a three‐necked flask equipped with a condenser. Under a nitrogen atmosphere, TCBA was added dropwise to the spermine solution, and the reaction was maintained at 50 °C. Prior to gelation, an excess of an amine‐based quenching agent was added to terminate the reaction. The resulting mixture was diluted with 20 mL of water, the pH was adjusted to 4.0 using 10 m HCl, and the solution was dialyzed against water (MWCO: 3500 Da) to remove unreacted spermine.

### 4.5 Determination of Proton Buffering Capacity

The proton buffering capacity of the polymer was quantified via acid‐base titration following established protocols [62]. Briefly, 1 mg of DSSP was dissolved in 2 mL of 150 mM NaCl solution (0.4 mg/mL). The polymer solution was initially adjusted to pH 10.0 using 0.1 M NaOH and then titrated stepwise to pH 3.0 with 0.1 M HCl under continuous stirring. After equilibrating at room temperature for 3 min, pH values were recorded using a calibrated pH meter (Mettler Toledo FE28). Spermine (10 mM in 150 mM NaCl) and 150 mM NaCl alone were used as positive and negative controls, respectively.

The buffering capacity (%) between pH 7.4 and 5.1 was defined as the percentage of protonatable amine groups and calculated using the appropriate formula:

$$\mathrm{Buffering}\;\mathrm{capacity}\;\left(\%\right)=\frac{\Delta V_{HCl}\times 0.1\;M}{N}\times 100\%$$

where: Δ*V*_HCl_ represents the volume (L) of 0.1 M HCl required to decrease the pH from 7.4 to 5.1.

*N* is the total moles of protonatable amine groups in DSSP or spermine.

### 4.6 Characteristics

#### 4.6.1 Instruments

The pure product was vacuum‐dried and characterized by nuclear magnetic resonance (NMR) spectroscopy using a Bruker AVANCE‐600 instrument. IR spectra of DSSP, SP, and TCBA were recorded using a Fourier transform infrared spectrometer (Nicolet, Thermo Fisher). TEM imaging was performed on a Talos F200X transmission electron microscope (Thermo Fisher). The hydrodynamic diameter and zeta potential of the samples were measured using a Zetasizer Nano ZS (Malvern Instruments).

#### 4.6.2 In Vitro Smart‐Responsive Release of siRNA

To evaluate the release profile of siRNA from siRNA/DSSP NPs, 1 mL of siRNA/DSSP NPs suspension (0.5 mg/mL siRNA) was incubated in phosphate buffer containing 10 mM GSH and 1 mM H_2_O_2_ at 37 °C with gentle agitation (150 rpm). At predetermined intervals (0, 2, 4, 8, 12, and 24 h), 500 µL aliquot were collected and subjected to ultracentrifugation at 40 000×g for 30 min. The supernatant was immediately replaced with an equal volume of fresh buffer to maintain sink conditions. The cumulative release of FAM‐siRNA was quantified by measuring the fluorescence intensity (λex/λem = 488/520 nm) using a fluorescence microplate reader (BioTek Synergy H1).

### 4.7 Cell Study

#### 4.7.1 Cell Culture

A2780, HCT116 and ID8 cells were obtained from the Cell Bank of the Chinese Academy of Science. All cell lines were routinely tested and confirmed to be free of mycoplasma contamination. A2780 and ID8 cells were cultured in Dulbecco's Modified Eagle's Medium (DMEM; Gibco, Waltham, MA, USA) supplemented with 10% certified heat‐inactivated fetal bovine serum (FBS; Gibco), 100 U/mL penicillin, and 100 µg/mL streptomycin, at 37 °C in a humidified atmosphere containing 5% CO_2_. HCT116 cells were maintained in RPMI 1640 medium (Gibco) supplemented with 10% FBS. Human umbilical vein endothelial cells (HUVECs) were purchased from PromoCell (C‐12208) and cultured in Endothelial Cell Growth Medium 2 (PromoCell, C‐22011). HUVECs were used only up to passage six. All cells were maintained in a humidified incubator at 37 °C with 5% CO_2_.

#### 4.7.2 Evaluation of Cytotoxicity of DSSP

A2780 cells were seeded in 96‐well plates at a density of 1 × 10^4^ cells per well to assess the cytotoxicity of DSSP and PSP‐Control. After 24 h of incubation with the polymers, 10 µL Cell Counting Kit‐8 (CCK‐8) reagent (NMPM Biotech, C6005) was added to each well, followed by an additional 4‐h incubation at 37 °C. For cells subjected to various treatments, after 48 h of culture, 10 µL of CCK‐8 solution was added and incubated for 2 h. Absorbance was measured at 450 nm using a microplate reader.

#### 4.7.3 Intracellular Uptake and Distribution Assay

To evaluate the intracellular uptake of siRNA, Cy3‐labeled siRNA (red fluorescence) was used to prepare fluorescent siRNA/DSSP@DSPE‐PEG and siRNA/DSSP@DSPE‐PEG‐FA nanoparticles. ID8 cells were seeded in confocal culture dishes at a density of 1×10^4^ cells/dish and cultured for 24 h at 37 °C and 5% CO_2_. Subsequently, the cells were co‐incubated with siRNA/DSSP@DSPE‐PEG and siRNA/DSSP@DSPE‐PEG‐FA for 2 and 6 h under the same conditions. After incubation, excess nanoparticles were removed by washing with PBS, followed by a further 20‐minute incubation in RPMI‐1640 medium containing Lyso‐Tracker Green. The cells were then washed with PBS, stained with Hoechst for 30 min to visualize the nuclei, and washed three times with PBS. The intracellular distribution of siRNA/DSSP@DSPE‐PEG‐FA at 6 h was observed using CLSM.

#### 4.7.4 Flow Cytometric Analysis of Cellular Uptake

To quantitatively evaluate nanoparticle internalization, ID8 cells were treated with Cy3‐labeled siRNA/DSSP@DSPE‐PEG or siRNA/DSSP@DSPE‐PEG‐FA (100 nM siRNA equivalent) for 1, 3, or 6 h under standard culture conditions (37 °C, 5% CO_2_). Following incubation, cells were washed with PBS, trypsinized, harvested, and resuspended in PBS supplemented with 1% BSA. The fluorescence intensity (λex/λem = 561/580 nm) of 10 000 events per sample was measured using a BD FACSCanto II flow cytometer (BD Biosciences).

#### 4.7.5 Evaluation of Transfection Efficiency

To assess the transfection efficacy of siLin28b/DSSP@lip‐PEG and silin28b/DSSP@lip‐PEG‐FA, A2780, ID8, HCT116, and CT26 cells were seeded in 6‐well plates at a density of 5×10^4^ cells per well. Cells were treated with siLin28b/DSSP@lip‐PEG or siLin28b/DSSP@lip‐PEG‐FA for 48 h, after which target protein expression was evaluated by Western blot analysis. In addition, the knockdown efficiency of siLIN28B on the LIN28B gene in HCT116 cells was determined by quantitative real‐time polymerase chain reaction (qRT‐PCR) assay. RNA extraction and qRT‐PCR were performed as follows: Total RNA was extracted from the cells using the EZb kit according to the manufacturer's instructions. Total RNA was extracted using the EZb kit following the manufacturer's protocol. Complementary DNA (cDNA) was synthesized from 1 µg of total RNA using the HiScript III RT SuperMix (Vazyme, #R323‐01). qRT‐PCR was performed with ChamQ Universal SYBR qPCR Master Mix (Vazyme, #Q711‐02) on a QuantStudio system. All primers were synthesized by Huada Gene. The primer sequences used in this study were as follows:

Human LIN28B_forward: 5′‐AAGAAGACCCAAAGGGAAGACAC‐3′;
Human LIN28B_reverse: 5′‐CACTTCTTTGGCTGAGGAGGTAG‐3′;
Mouse LIN28B_forward: 5′‐ CGAGATGGCTCAGTGGGTTC‐3′;
Mouse LIN28B_reverse: 5′‐GGCTCTTCACCTTTGCTTGC‐3′;
Human GAPDH_forward: 5′‐ACACCATGGGGAAGGTGAAG‐3′;
Human GAPDH_reverse: 5′‐AAGGGGTCATTGATGGCAAC‐3′;
Mouse actin_forward: 5′‐ ATCTTCCGCCTTAATACT‐3′;
and Mouse actin_reverse: 5′‐ GCCTTCATACATCAAGTT‐3′.

#### 4.7.6 Immunostaining for Cultured Cells

HUVECs were seeded into 6‐well plates at a density of 1×10^6^ cells per well and stimulated with vehicle, LPS, TNF‐α, or IL‐6. After 24 h, RNA and protein were collected, and Lin28b mRNA and protein expression levels were assessed by qPCR and Western blot, respectively.

To assess cell‐cell junctions, LipoRNAi Transfection Reagent (C0535, beyotime) was mixed with siLin28b or control siRNA (catalog number) in Reduced Serum Medium (catalog number) according to the manufacturer's protocol. The mixture was then incubated with HUVECs for 6 h at 37 °C in a 12‐well plate, with a working concentration of 80 pmol siRNA per well. After incubation, fresh medium was added, and cells were cultured for an additional 24 h before treatment with 10 nmol BMN673 or vehicle for another 24 h. Finally, cells were stimulated with 100 ng/mL TNF‐α (210‐TA‐100; R&D) or 1 µg/mL LPS (L2630, Sigma‐Aldrich) for 24 h prior to immunostaining analysis. Cells were fixed with 4% paraformaldehyde for 10 min at 4 °C, washed with PBS, and then permeabilized and blocked for 1 h at room temperature using 10% donkey serum (935; Absin) and 0.5% Triton‐X100 in 1×PBS. anti‐VeCadherin (1:200, 2500, Cell Signaling Technology) antibodies were diluted in a blocking solution containing 10% donkey serum, 1% BSA (A600332; Sangon), and 0.2% Tween‐20 (A600560; Sangon) in 1×PBS. Cells were incubated with primary antibodies overnight at 4°C. After three washes with 0.3% Tween‐20 in 1×PBS (30 min each), cells were incubated with secondary antibodies diluted in the same blocking solution for 1 h at room temperature. Following another three washes, samples were mounted using ProLong mounting medium (P36961; Invitrogen). Quantitative analysis of Ve‐cadherin‐expressing cells was performed using the ratio of the Ve‐cadherin‐positive area to the total nuclear number for normalization.

#### 4.7.7 Evaluation of Cytokine‐Induced Permeability Changes in HUVEC Monolayers

HUVECs were seeded at a density of 1×10^5^ cells per well in 24‐well Transwell inserts and cultured for 4 days to achieve full confluency and form an endothelial monolayer. The endothelial growth medium was refreshed every two days. For TNF‐α treatment, HUVECs were stimulated with TNF‐α (100 ng/mL) in the presence or absence of TNF‐α neutralizing antibody (15 µg/mL) for 6 h, followed by the addition of FITC‐dextran (1 mg/mL in HBSS) for to the upper chamber for 30 min. In the end, medium from the lower chamber was collected, and fluorescence intensity measurement. For IL‐6 treatment, HUVECs were exposed to IL‐6 (100 ng/mL) with or without IL‐6 neutralizing antibody (5 µg/mL) for 12 h, followed by incubation with FITC‐dextran (1 mg/mL in HBSS) for 60 min. Fluorescence intensity was quantified using same approach described above.

### 4.8 Animals Study

#### 4.8.1 Animals

C57BL/6 or Balb/c nude mice (6–8 weeks old) were obtained from Beijing Vital River Laboratory Animal Technology. All animals were housed under specific pathogen‐free (SPF) conditions, with free access to food and water, at a controlled temperatures of 22 ± 2 °C and a 12‐h light/dark cycle. All animal experiments were conducted in accordance with institutional ethical guidelines and were approved by the Animal Care and Use Committee of Shanghai Jiao Tong University (Approval No. A2023097‐003).

#### 4.8.2 Biodistribution In Vivo

To evaluate the in vivo biodistribution of DSSP@lip‐PEG and DSSP@lip‐PEG‐FA, we conducted a fluorescence imaging experiment. Female Balb/c nude mice were subcutaneously injected with HCT116 cells until the tumor volume reached approximately 400 mm^3^. Following intravenous administration of DSSP@lip‐PEG and DSSP@lip‐PEG‐FA (containing Cy5.5‐labeled siRNA at a dose of 10 nmol), fluorescence imaging was carried out at 4‐, 12‐, 24‐ and 48‐h post‐injection using the Chemiluminescence/Fluorescence Imaging Analysis System (PerkinElmer, USA). At 48 h postinjection, the mice were sacrificed, and major organs (heart, liver, spleen, lungs, and kidneys), as well as tumors, were harvested and imaged.

#### 4.8.3 Antitumor Analysis

To investigate the combinatorial effects of siLin28b and BMN673, clonogenic assays were performed on HCT116 and A2780 cells. Specifically, 500 cells per well were seeded into 6‐well plates and allowed to adhere for 3 days. Afterward, cells were treated with varying concentrations of siLin28b and BMN673 at different ratios for a defined period. Colonies were stained, imaged, and quantified using ImageJ software to assess cytotoxicity and proliferation inhibition. For in vivo evaluation, a subcutaneous xenograft model was established by injecting HCT116 cells (5×10^6^ cells in 0.1 mL PBS) into the right flank of mice. When the tumors reached an average volume of approximately 100 mm^3^, the mice were randomized into four treatment groups: vehicle control, siLin28b alone (10 ng/mouse, i.p.), BMN673 alone (0.33 mg/kg, i.g.), and the siLin28b+BMN673 combination. Tumor volumes and body weights were measured every other day to monitor therapeutic efficacy and potential toxicity.

#### 4.8.4 Anti‐Malignant Ascites In Preclinical OC Model

To evaluate the therapeutic effects of siLin28b combined with BMN673 in a preclinical OC model, C57BL/6 mice were intraperitoneally injected with 1×10^7^ ID8 cells [63, 64]. On day 10 post‐injection, baseline abdominal fluorescence was assessed using the IVIS Lumina II optical imaging system. The mice were then randomized into four treatment groups: vehicle control, siLin28b alone (i.p.: 10 nmoL/mouse), BMN673 alone (i.g.: 0.33 mg/kg) [65, 66], and the combination of siLin28b+BMN673. Tumor progression was monitored using the IVIS Lumina II system. Survival analysis was performed by measuring abdominal circumference, with the endpoint defined as the point at which the abdominal circumference exceeded twice the normal size of healthy mice. On day 45, abdominal distension was quantified using a Multimodal Small Animal Ultrasound/Photoacoustic Imaging System (VEVO LAZR‐X, Fujifilm VisualSonics, USA). Upon euthanasia on day 45, malignant effusion samples were collected for cytokine profiling using ELISA kits.

#### 4.8.5 Anti‐Malignant Ascites in Non‐Ovarian MA Models

To evaluate the ascites‐suppressing effect of siLin28b combined with BMN673 in a non‐ovarian cancer setting, we established a CT26 ascites model. A total of 1 × 10^6^ CT26 cells suspended in 100 µL PBS were injected intraperitoneally into BALB/c mice, and ascites formation became apparent around day 5 post‐injection. The mice were then randomized into two treatment groups: vehicle control and siLin28b plus BMN673. On day 30, abdominal distension and ascites accumulation were assessed and quantified.

#### 4.8.6 Regulation of Immune Cells in Ascites Suppression

To investigate the role of immune cells in ascites suppression, we combined anti‐CD8 treatment with the siLin28b+BMN673 administration regimen described in Section 4.7.4 in a preclinical ovarian cancer model, in order to assess whether CD8^+^ T cell depletion affects the ability of siLin28b+BMN673 to inhibit ascites formation. Additionally, based on single‐cell analysis, neutrophils or macrophages were selectively depleted to evaluate their individual contributions to ascites suppression.

#### 4.8.7 Multi‐Factor Detection and Analysis

To elucidate the pathogenic mechanisms underlying malignant effusion formation, cytokine levels in paired serum and malignant effusion samples were quantitatively analyzed using Luminex xMAP‐based multiplex immunoassay. The capture antibody carrying biomarkers is covalently coupled to magnetic beads. After the coupled beads are combined with the sample containing the target biomarker (for 30 min at a rotational speed of 800 revolutions per minute), several washing steps are performed to remove the unbound proteins. The beads are adsorbed by a powerful magnetic rack for at least 1 min during this process. Subsequently, the biotinylated detection antibody is added to generate a sandwich complex (incubated for 30 min at 800 rpm). Then, the streptavidin‐phycoerythrin (SA‐PE) conjugate is introduced to form the final detection complex (incubated for 10 min at 800 rpm). Finally, the sample is detected using a relevant instrument. The resulting data are processed using Python, and data visualization is performed using Matplotlib.

#### 4.8.8 ELISA Analysis of Ascites

On day 45, ascites fluid was collected from ovarian cancer–bearing mice receiving the different treatments. According to the instructions provided with each ELISA kit, the levels of the indicated factors were measured to determine whether their concentrations changed in response to treatment.

#### 4.8.9 Evans Blue Permeability Assay

To assess vascular permeability in mice, 150 µL of Evans blue solution (30 mg/kg, dissolved in saline) was slowly injected via the tail vein 10 min before sacrifice. After anesthesia, the thoracic cavity was opened, and a cannula was inserted into the right ventricle. This was followed by perfusion with PBS at a rate of 8 mL/min for 5 min to flush out residual Evans blue from the vasculature. Finally, the organs were carefully dissected and rinsed with PBS.

#### 4.8.10 Immunostaining for Mesenterium and Peritoneum

Mesenterium and peritoneum samples were fixed in 4% PFA overnight at 4 °C, then immersed in 30% sucrose for 24 h before cryopreservation in optimal cutting temperature compound at −80 °C. Samples were rinsed with PBS, permeabilized, and blocked in 10% donkey serum (935; Absin) and 5% Triton‐X100 in 1× PBS overnight at 4 °C. Primary antibodies were diluted in 10% donkey serum, 1% BSA (A600332; Sangon), and 0.2% Tween‐20 (A600560; Sangon) in 1× PBS, and samples were incubated with them for 2 days at 4 °C. After three washes with 0.3% Tween‐20 in 1× PBS (2 h each), samples were incubated with secondary antibodies diluted in the same blocking solution for 2 days at 4 °C. Following three additional washes, samples were cleared using Rapiclear 1.49(SUNJin Lab, RC149001) and imaged using a spinning disk confocal microscopy (Andor, Dragonfly). Primary antibodies used in this study: Anti‐CD31 antibody (1:200, MA3105, Thermo Fisher Scientific) Anti‐TER‐119 antibody (1:100, 557915, BD Biosciences).

#### 4.8.11 Evaluation of Biocompatibility In Vivo

After completing the treatment, major organs (heart, liver, spleen, lung, and kidney) and tumors were collected for histological analysis. Subsequently, H&E staining was performed to assess any potential damage to the organs caused by siLIN28B+BMN673. Additionally, serum biochemical markers, including aspartate alanine aminotransferase (ALT), blood urea nitrogen (BUN), total bilirubin (TBIL), creatinine (CREA), uric acid (UA), and aspartate aminotransferase (AST), were analyzed to assess the safety profile of each formulation. Additionally, TUNEL staining was performed on the harvested organs to evaluate apoptosis, and immunohistochemical analysis of the heart was conducted to assess cardiac troponin levels, providing a comprehensive evaluation of treatment safety. ELISA was performed to measure the levels of anti‐PEG antibodies and IL‐6 levels in the serum, providing an additional assessment of the safety of siLin28b/DSSP@lip‐PEG‐FA.

### 4.9 Statistical Analysis

Statistical analysis and data visualization were performed using GraphPad 9.0 software. All data are presented as mean ± standard deviation (SD). Statistical significance was defined as follows: *p*≥0.05, not significant (ns); ^*^ *p* < 0.05, ^**^ *p* < 0.01, and ^***^ *p* < 0.001 were considered statistically significant.

## Author Contributions

Y.F., Q.S., Y.L., J.Z., and Z.X. performed the experiments, analyzed the results and wrote the manuscript. R.H., Q.L., and F.S. assisted with animal study. Q.C. provided patient samples and research materials. Y.W. and G.Z. assisted with cell‐based assays and the generation of cartoon diagrams. Y.L. and Z.Z. assisted with bioinformatic analysis and provided consultation. J.S. and J.H. designed the experiments, analyzed the results and wrote the manuscript.

## Funding

This work was supported by General Program of National Natural Science Foundation of China (Nos. 82472752 and 81972667 to J.S., 82203009 to Q.S., and 62131009 to Q.C.), National Key R&D Program of China (2021YFC2701103 to J.S.), and the Center for High Performance Computing at Shanghai Jiao Tong University.

## Conflicts of Interest

The authors declare no conflict of interest.

## Supporting information




**Supporting File 1**: advs73768‐sup‐0001‐SuppMat.docx.

**Supporting File 2**: advs73768‐sup‐0002‐TableS1.xlsx.

## Data Availability

All sequencing data generated in this study have been deposited in the Gene Expression Omnibus (GEO) database under accession numbers GSE293216 and GSE295677.
