# Supplementary material for: PARPi Combining Nanoparticle LIN28B siRNA for the Management of Malignant Ascites
Source: Adv Sci (Weinh). 2026 Jan 22;13(16):e10547. doi: 10.1002/advs.202510547 (PMC13042977; doi:10.1002/advs.202510547)
Supplement: Supplementary file 1 — Supporting File 1: advs73768‐sup‐0001‐SuppMat.docx. [file ADVS-13-e10547-s001.docx]

**Supporting Information**

**PARPi Combining Nanoparticle LIN28B siRNA for the Management of Malignant Ascites**

*Yan Fang ^1,2,3^****^#^****, Qian Shen ^4^****^#^****, Yao Lin ^5^****^#^****, Jing Zhu ^6^****^#^****, Xiaolan Zhu ^6^****^#^****,* *Rui Huang ^1,2,3^,* *Yijia Wu ^1,2^, Feiyang Shen ^1,2^, Qian Li ^1,2,3^, Guopei Zheng ^1,2,3^, Zhe Zhang ^1,2,3^, Qian Chu ^4*^, Junhao Hu ^6*^ and Jianfeng Shen ^1,2,3*^*

1. Department of Ophthalmology, Ninth People’s Hospital, Shanghai Jiao Tong University School of Medicine, Shanghai, 200025, China.

2. Shanghai Key Laboratory of Orbital Diseases and Ocular Oncology, Shanghai, 200025, China.

3. Institute of Translational Medicine, National Facility for Translational Medicine, Shanghai Jiao Tong University, Shanghai, 200240, China.

4. Department of Oncology, Tongji Hospital, Tongji Medical College, Huazhong University of Science and Technology, Wuhan 430030, China.

5. Department of Gastrointestinal Surgery, Union Hospital, Tongji Medical College, Huazhong University of Science and Technology, Wuhan 430022, China.

6. Interdisciplinary Research Center on Biology and Chemistry, Shanghai Institute of Organic Chemistry, Chinese Academy of Sciences, Shanghai, China; University of Chinese Academy of Sciences, Beijing, China.

#These authors contributed equally to this article.

*Correspondence should be addressed to Jianfeng Shen (jfshen@shsmu.edu.cn), Junhao Hu (jhhu@sioc.ac.cn) and Qian Chu (qianchu@tjh.tjmu.edu.cn)


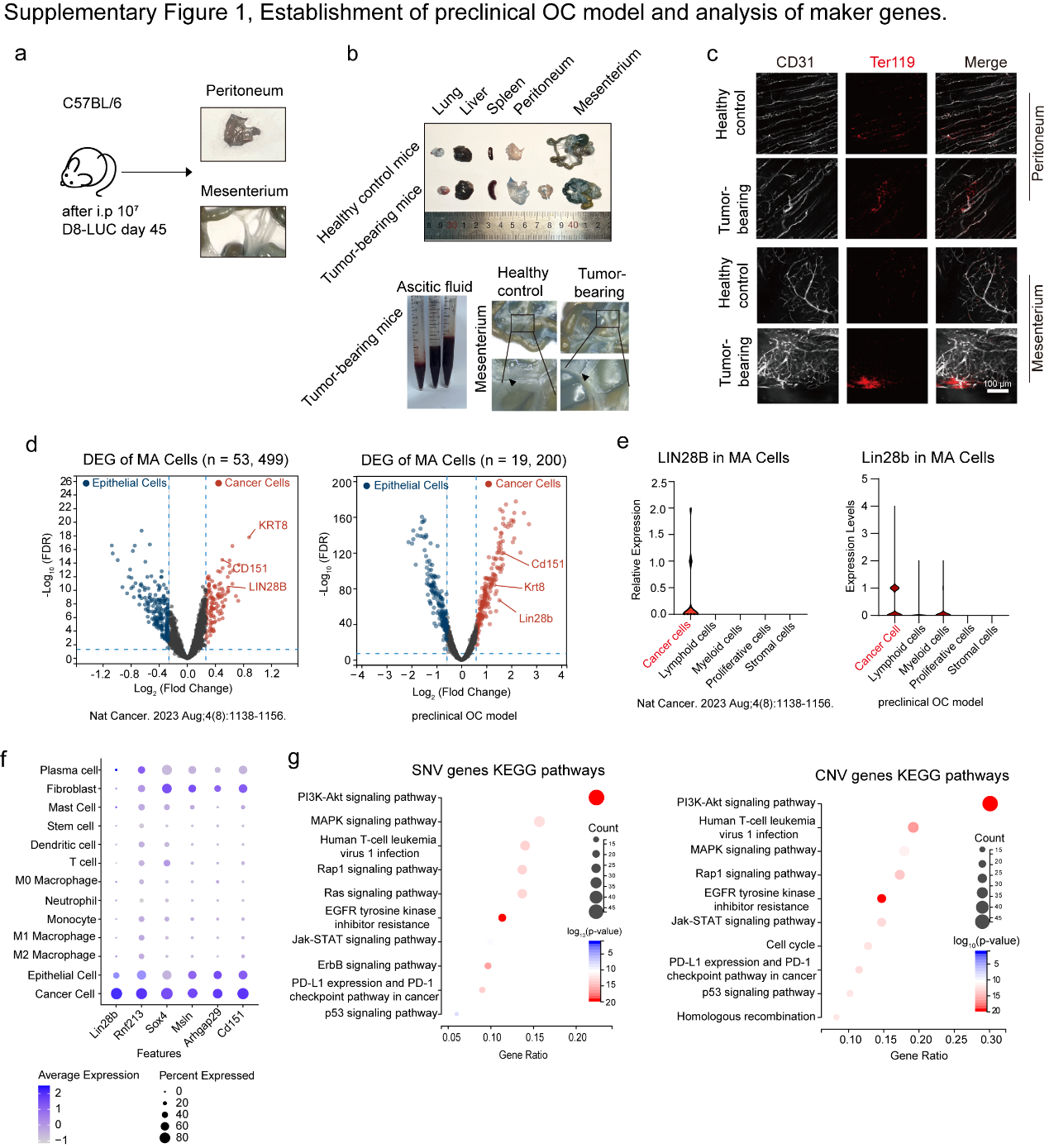


**Figure S1.** Establishment of preclinical OC model and analysis of maker genes. (a) The establishment of the preclinical OC model. (b) Characterization of the features of preclinical OC model. (c) Immunohistochemical analysis of CD31 and Ter119 to determine the mesenteric and peritoneal vascular permeability. (d) DEG analysis of ascites cells from both the preclinical OC model and OC patients (Nat Cancer. 2023). (e) Quantitative analysis of LIN28B expression across various cell types from both the preclinical OC model and OC patients (Nat Cancer. 2023). (f) Dot plots for the expression of six candidate genes in each cell type. (g) DEG analysis for SNV gene mutations and CNV gene mutations in serous effusions from cancer patients.


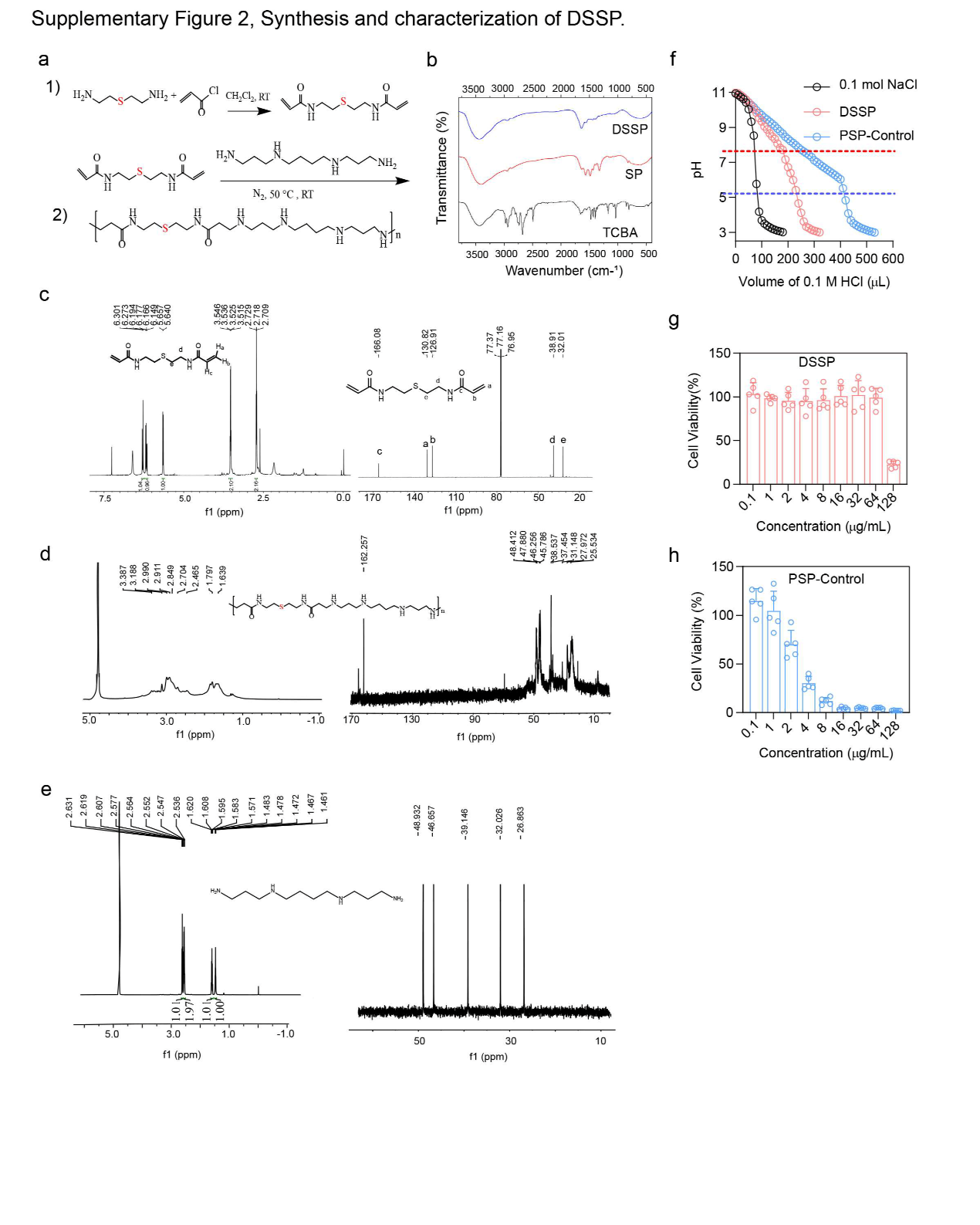


**Figure S2.** Synthesis and characterization of DSSP. (a) Synthetic route of DSSP. (b) FT-IR spectroscopy analysis of SP, TCBA, and DSSP. (c) ^1^H- and ^13^C-NMR spectra of TCBA. (d) ^1^H- and ^13^C-NMR spectra of DSSP. (e) ^1^H- and ^13^C-NMR spectra of SP. (f) pH-dependent titration curves of DSSP in 150 mM NaCl aqueous solution (0.1 M HCl titration). (g) Cytotoxicity evaluation of DSSP and (h) PSP-Control.


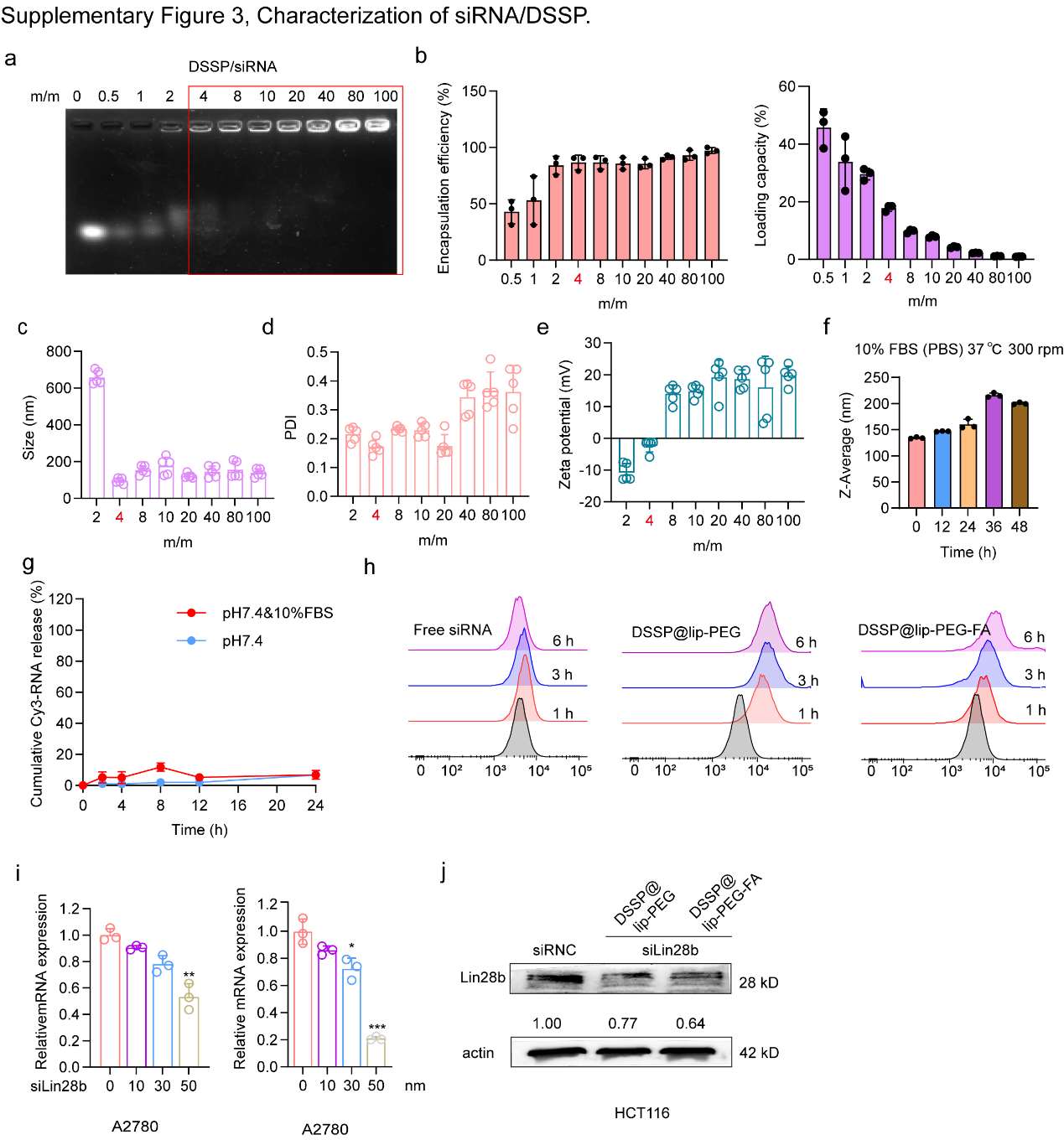


**Figure S3.** Characterization of siRNA/DSSP@lip-PEG-FA. (a) Gel retardation assay of condensing eﬃcacy of siRNA by the DSSP at various mass ratios. (b) Encapsulation efficiency and loading capacity of siRNA. Mean diameter (c), PDI (d) and zeta potential (e) of siRNA/DSSP NPs at various ratios of DSSP to siRNA. (f) Particle size of siRNA/DSSP@lip-PEG-FA incubated in serum at 37 °C with shaking at 300 rpm for 0–48 hours. (g) Cumulative release profile of siRNA from siRNA/DSSP@lip-PEG-FA under physiological conditions. (h) Flow cytometry analysis for intracellular uptake of Cy3-siRNA in ID8 cells. (i) Real-time PCR analysis of LIN28B in A2780 cells treated with siLIN28B/DSSP@lip-PEG or siLIN28B/DSSP@lip-PEG-FA. (j) Immunoblotting of LIN28bB protein in HCT116 cells treated with siLIN28B/DSSP@lip-PEG or siLIN28B/DSSP@lip-PEG-FA. Data are mean ± s.d. (n = 3, unpaired t-test). *P < 0.05, **P < 0.01, ***P < 0.001.


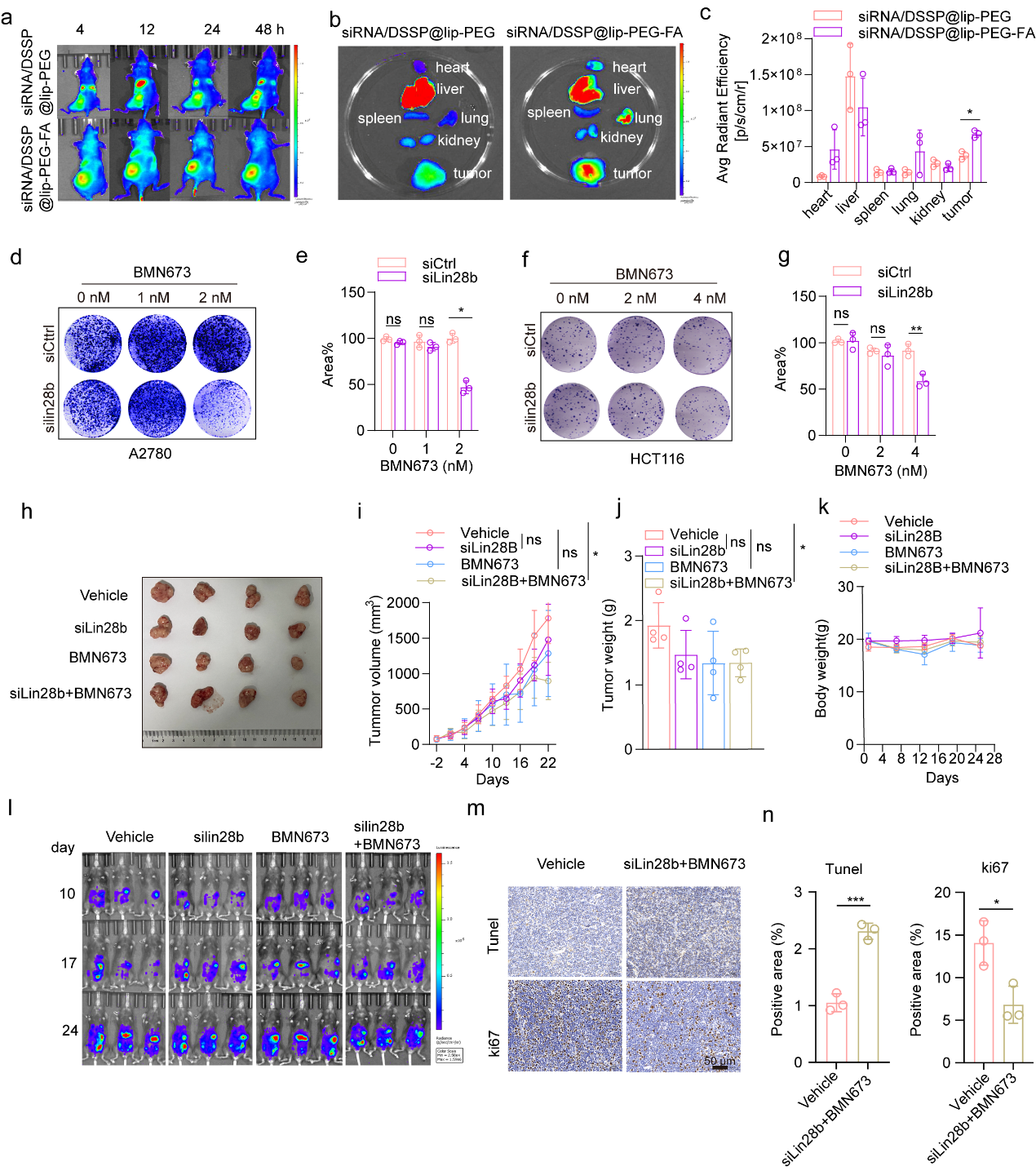


**Figure S4.** *In vivo* tumor targeting and anti-tumor efficacy of LIN28B siRNA and BMN673. (a) Images of HCT116-tumor bearing mice after treatment with DSSP@lip-PEG or DSSP@lip-PEG-FA at different time points. (b) NIR fluorescence imaging of the harvested organs and tumors at 48 h post-administration. (c) Quantitative analysis of Cy5.5 fluorescence intensity in organs and tumors of mice (n=3, unpaired t-test). (d) Representative images and quantitative analysis (e) of cloning formation assay in A2780 under the treatment of LIN28B siRNA and BMN673 (n=3, unpaired t-test). (f) Representative images and quantitative analysis (g) of cloning formation assay in HCT116 under the treatment of LIN28B siRNA and BMN673 (n=3, unpaired t-test). Representative images (h) and tumor growth curves (i) of HCT116 tumors treated with vehicle, siLIN28B, BMN673 and siLIN28B+BMN673 (n=5, ANOVA was used for multiple comparisons among three or more groups). (j) The tumor weight of HCT116 tumor-bearing mice receiving various treatments (n=4, unpaired t-test). (k) Body weight of HCT116 tumor-bearing mice after respective treatments (n=4). (l) *In vivo* bioluminescence imaging of tumor-bearing mice treated by vehicle, siLIN28B, BMN673 or siLIN28B+BMN673 at day 10, 17, and 24 post-inoculation of murine ovarian cancer ID8 cells (n=3). (m) IHC staining of tumor tissues (n=3). (n) Quantification of TUNEL- and Ki67-positive areas (n=3, unpaired t-test). All quantitative results are presented with mean ± SD. *P < 0.05, **P< 0.01, ***P < 0.001; ns, not significant.


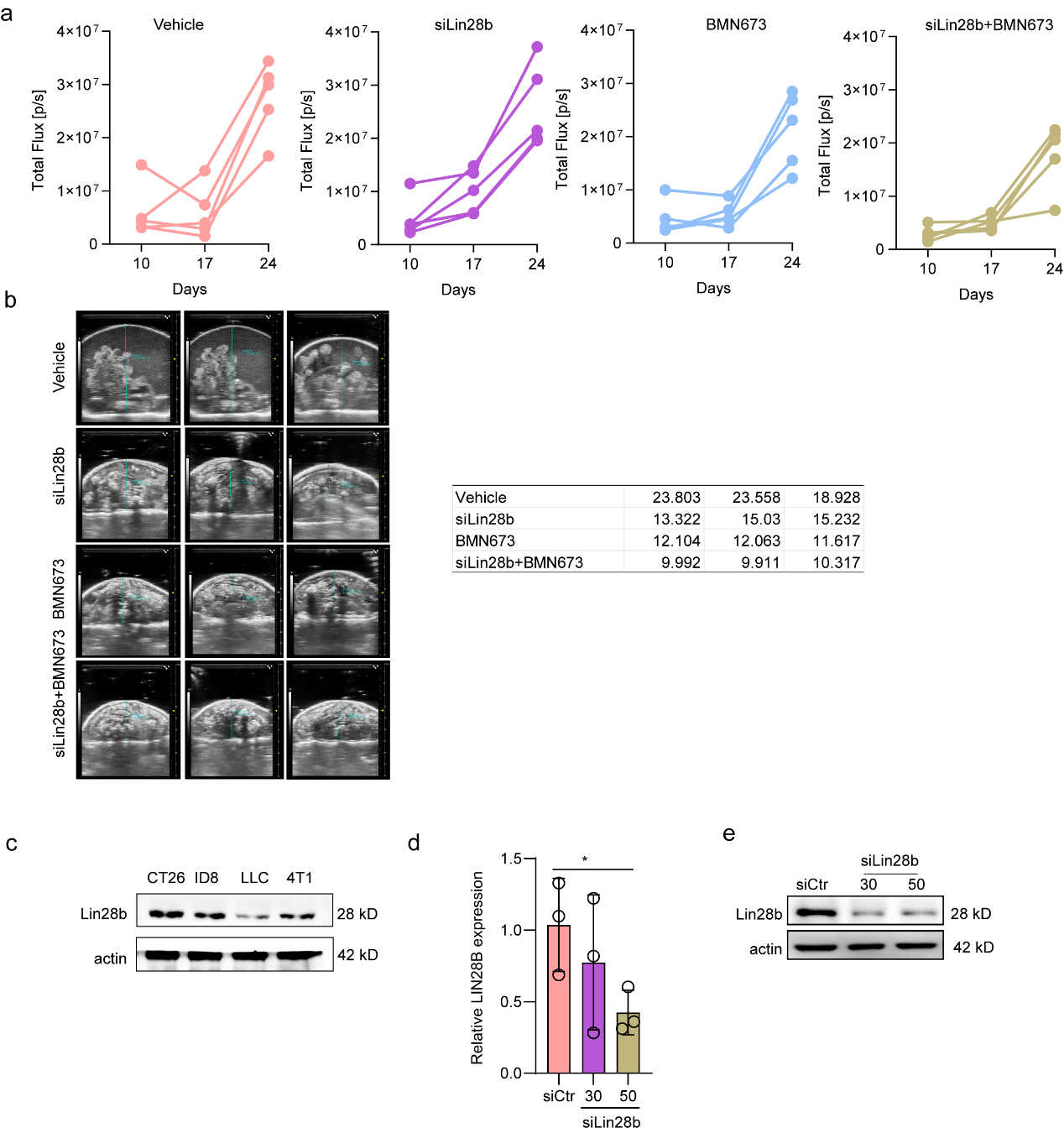


**Figure S5.** siLin28b+BMN673 to suppress tumor progression and reduce ascites formation. (a) Bioluminescent imaging quantification of total flux in tumor-bearing mice treated with vehicle, siLin28b, BMN673, or the combination of siLin28b and BMN673 at the indicated time points. Each line represents an individual mouse. (b) Representative ultrasound images showing abdominal ascites accumulation across treatment groups, including vehicle, siLin28b, BMN673, and siLin28b + BMN673. Quantification of ultrasound-measured ascites depth is shown on the right. (c) Western blot analysis of LIN28B expression across multiple tumor cell lines (CT26, ID8, LLC, 4T1). (d) Quantification of relative LIN28B expression following siLin28b transfection at the indicated doses compared to siControl (siCtr) (n=3, unpaired t-test). (e) Western blot validation of dose-dependent Lin28b knockdown by siLin28b (30 nM and 50 nM). Data are presented as mean ± s.d.; *P < 0.05.


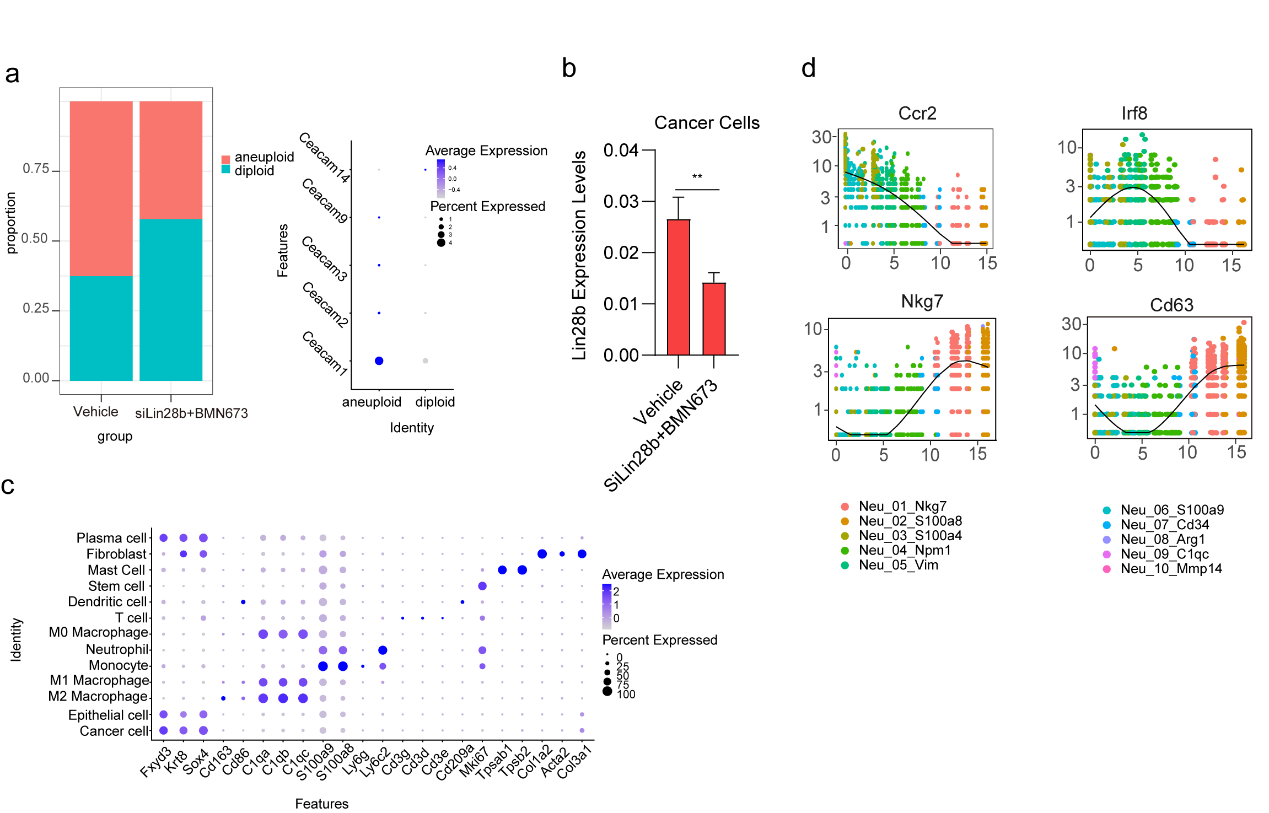


**Figure S6.** scRNA-seq analysis of ascites cells in preclinical OC model. (a) Assessment of tumor cell aneuploidy in vehicle and siLin28b + BMN673 groups using the Copykat algorithm. Proportion of aneuploid (red) and diploid (cyan) cells in each group. Expression of CEACAM family genes in aneuploid versus diploid cells, showing higher expression in aneuploid cells. (b) Quantitative analysis for Lin28b expression in ID8 preclinical OC models treated by vehicle or siLin28b+BMN673 (n=3, unpaired t-test). (c) Dot plots for the expression of marker genes in each cell type. (d) Genes jitterplot for Ccr2, Irf8, Nkg7 and Cd63 in neutrophil subtypes. **P < 0.01.


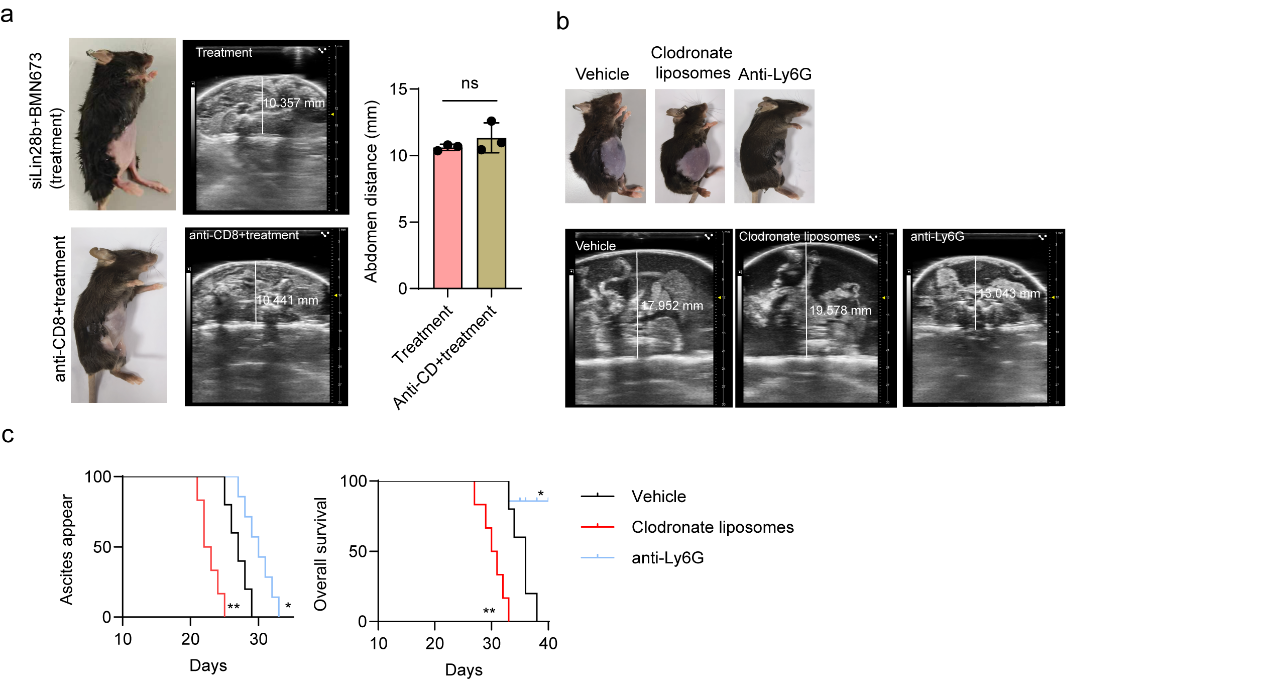


**Figure S7.** Depletion of CD8⁺ T cells or myeloid cells modulates MA. (a) Representative photographs and ultrasound images of mice bearing tumors following treatment with treatment or treatment + anti-CD8 antibody (n=3, unpaired t-test). (b) Representative photographs and ultrasound images of mice treated with vehicle, clodronate liposomes, or anti-Ly6G antibody (n=3). (c) Kaplan–Meier curves showing the time to ascites appearance (left) and overall survival (right) in mice treated with vehicle, clodronate liposomes, or anti-Ly6G antibody (n = 5, log-rank test). Data are mean ± s.d. *P < 0.05, **P < 0.01, ***P< 0.001; ns, not significant.


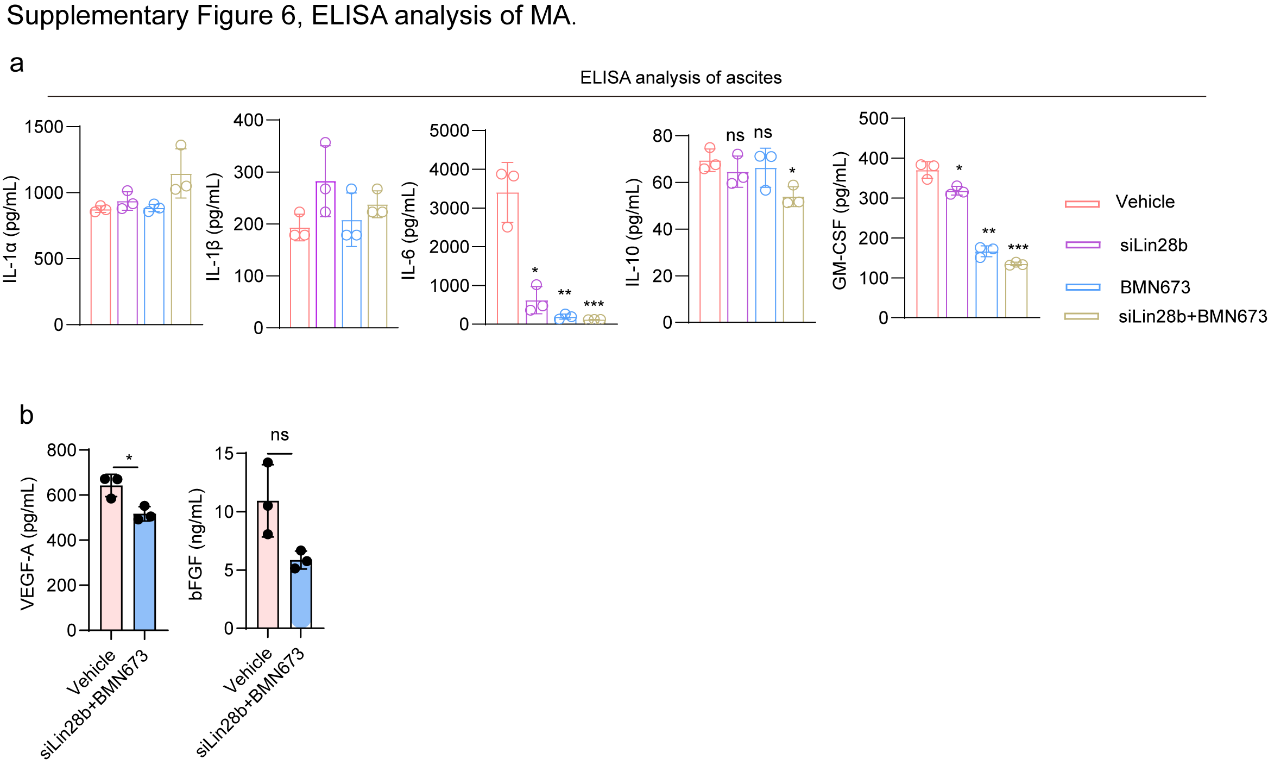


**Figure S8.** ELISA analysis of MA. (a). ELISA analysis of ascites fluid showing levels of IL-1α, IL-1β, IL-6, IL-10, and GM-CSF in mice treated with vehicle, siLin28b, BMN673, or the siLin28b + BMN673 combination. (b) Quantification of VEGFA and bFGF levels in ascites fluid. Data are mean ± s.d. (n = 3, unpaired t-test). *P < 0.05, **P < 0.01, ***P< 0.001; ns, not significant.


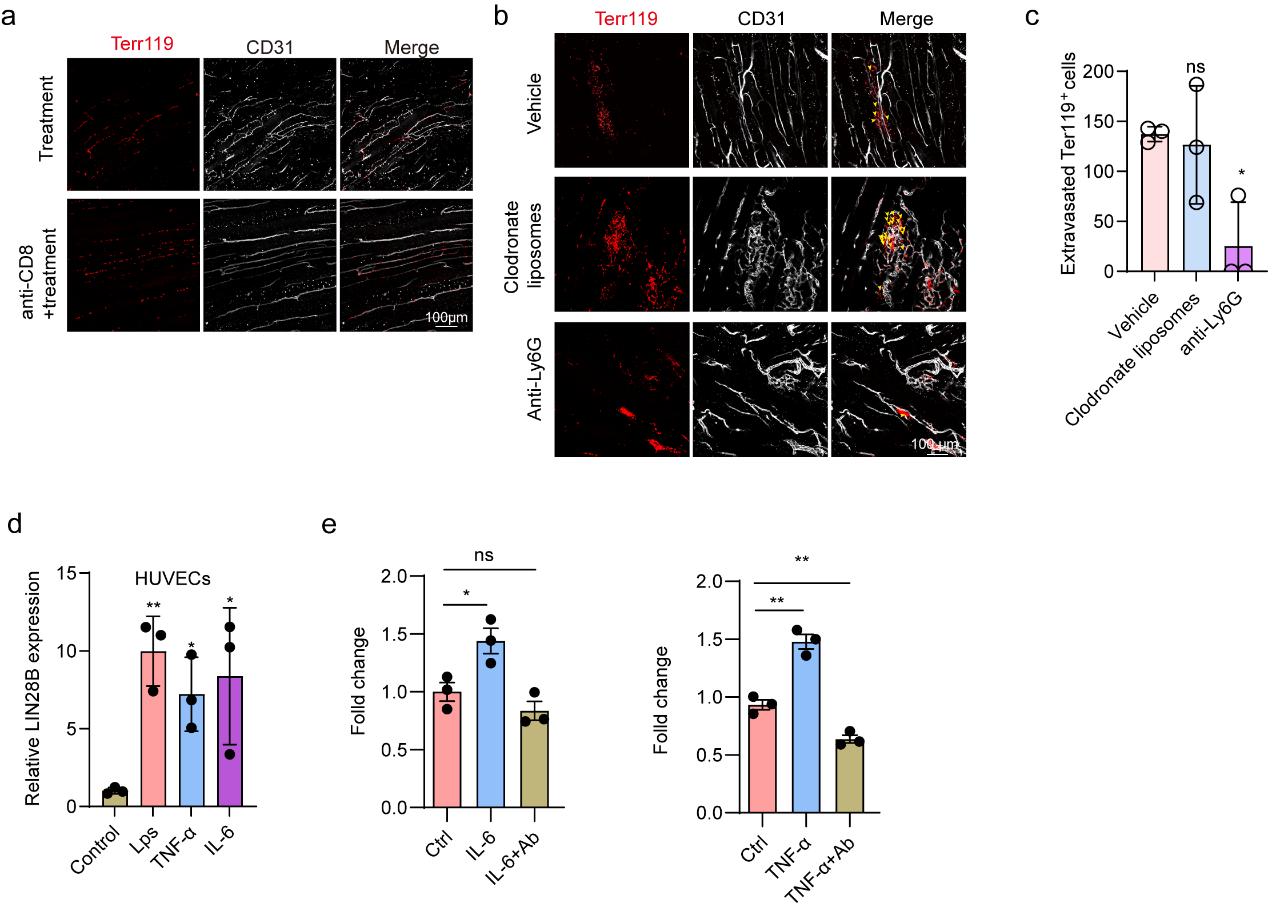


**Figure S9.** Immunofluorescence analysis of vascular integrity and inflammatory responses. (a) Immunofluorescence staining of peritoneal tissues across anti-CD8 plus treatment and treatment groups (scale bar: 100 μm). (b) Immunofluorescence staining of peritoneal tissues across different treatment groups (scale bar: 100 μm). (c) Quantification of extravasated Ter119⁺ cells per field. (d) Pro-inflammatory stimuli induce LIN28B expression in HUVECs. (e) Dextran-FITC leakage in HUVEC monolayers treated with TNFα or TNFα + Ab, and IL-6 or IL-6 + Ab. Data are mean ± s.d. (n = 3, unpaired t-test). *P < 0.05, **P < 0.01; ns, not significant.


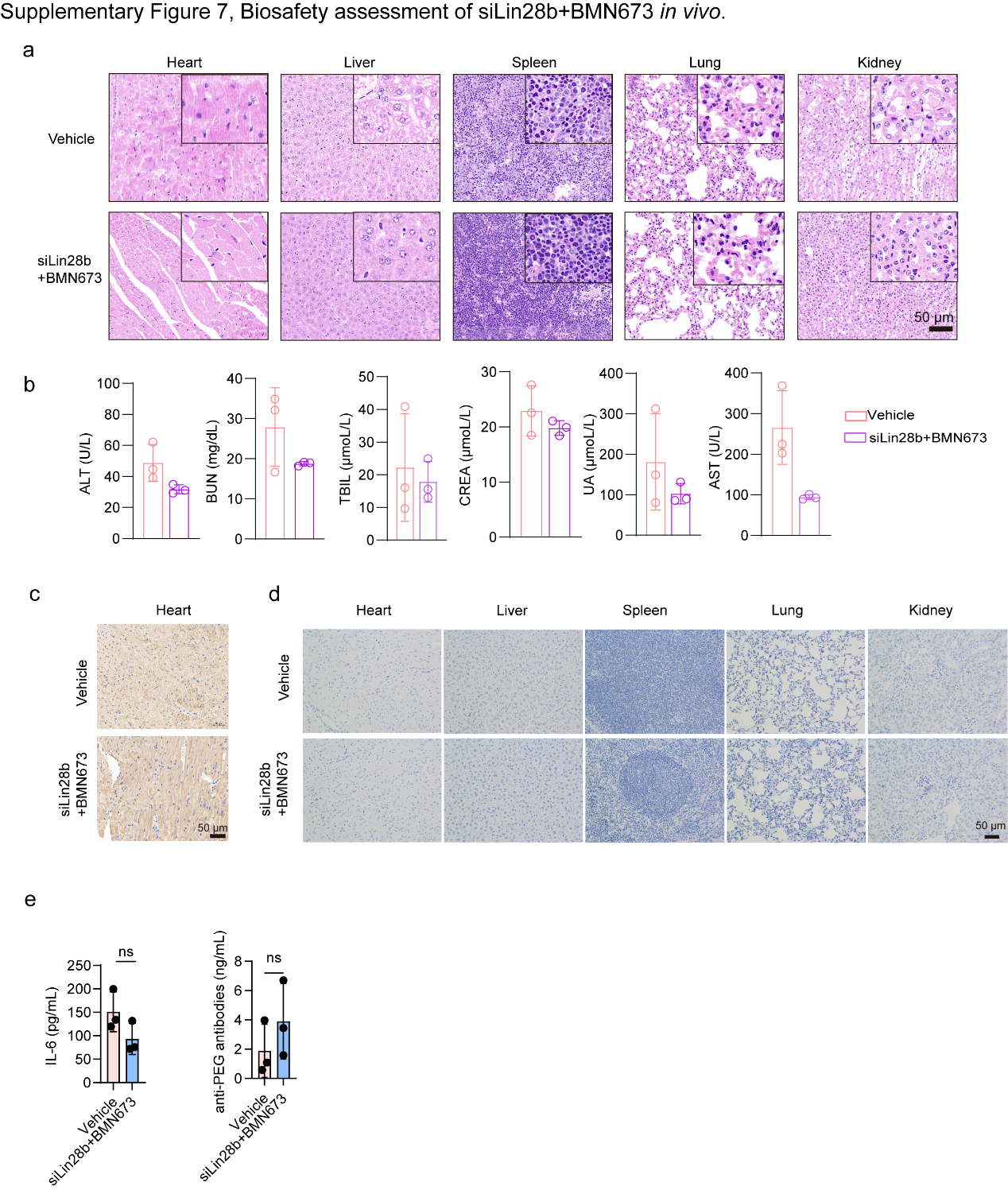


**Figure S10.** Biosafety assessment of siLin28b+BMN673 *in vivo*. (a) H&E staining of major organ tissues after 7 days of treatment with vehicle or siLin28b+BMN673. (b) Quantification of aspartate alanine aminotransferase (ALT), blood urea nitrogen (BUN), total bilirubin (TBIL), creatinine (CREA), uric acid (UA), and aspartate aminotransferase (AST) in the serum of mice after 7 days of treatment. (c) Immunohistochemical (IHC) staining of heart to evaluate cardiac troponin expression levels, reflecting potential myocardial injury. (d) Representative TUNEL staining images of major organs (heart, liver, spleen, lung, and kidney) to assess tissue apoptosis after different treatments. (e) Quantification of serum IL-6 levels and anti-PEG antibodies. Scale bar, 50 μm. All quantitative results are presented with mean ± SD (n = 3, unpaired t-test). ns, not significant.
